# Self-reported health behaviors, including sleep, correlate with doctor-informed medical conditions: data from the 2011 Health Related Behaviors Survey of U.S. Active Duty Military Personnel

**DOI:** 10.1186/s12889-018-5781-2

**Published:** 2018-07-11

**Authors:** Adela Hruby, Harris R. Lieberman, Tracey J. Smith

**Affiliations:** 1 Military Nutrition Division, US Army Research Institute of Environmental Medicine, Natick, MA 01760 USA; 2 Nutritional Epidemiology Program, Jean Mayer USDA Human Nutrition Research Center on Aging at Tufts University, Boston, MA 02111 USA

**Keywords:** Army, Navy, Air force, Marine corps, Coast guard, Cardiometabolic risk, Exercise, Sleep, Survey

## Abstract

**Background:**

Health behaviors and cardiometabolic disease risk factors may differ between military and civilian populations; therefore, in U.S. active duty military personnel, we assessed relationships between demographic characteristics, self-reported health behaviors, and doctor-informed medical conditions.

**Methods:**

Data were self-reported by 27,034 active duty military and Coast Guard personnel who responded to the 2011 Department of Defense Health Related Behaviors Survey. Multivariate linear and logistic regressions were used to estimate cross-sectional associations between (1) demographic characteristics (age, sex, service branch, marital status, children, race/ethnicity, pay grade) and self-reported behaviors (exercise, diet, smoking, alcohol, sleep); (2) demographic characteristics and doctor-informed medical conditions (hypertension, hypercholesterolemia, low high density lipoprotein (HDL) cholesterol, hyperglycemia) and overweight/obesity; and (3) behaviors and doctor-informed medical conditions.

**Results:**

Among respondents (age 29.9 ± 0.1 years, 14.7% female), females reported higher intake than men of fruit, vegetables, and dairy; those with higher education reported higher intakes of whole grains; those currently married and/or residing with children reported higher intake of starches. Older age and female sex were associated with higher odds (ORs 1.25 to 12.54 versus the youngest age group) of overweight/obesity. Older age and female sex were also associated with lower odds (ORs 0.29 to 0.65 versus male sex) of doctor-informed medical conditions, except for blood glucose, for which females had higher odds. Those currently married had higher odds of high cholesterol and overweight/obesity, and separated/divorced/widowed respondents had higher odds of high blood pressure and high cholesterol. Short sleep duration (< 5 versus 7–8 h/night) was associated with higher odds (ORs 1.36to 2.22) of any given doctor-informed medical condition. Strength training was associated with lower probability of high cholesterol, high triglycerides, and low HDL, and higher probability of overweight/obesity. Dietary factors were variably associated with doctor-informed medical conditions and overweight/obesity.

**Conclusions:**

This study observed pronounced associations between health behaviors—especially sleep—and medical conditions, thus adding to evidence that sleep is a critical, potentially modifiable behavior within this population. When possible, adequate sleep should continue to be promoted as an important part of overall health and wellness throughout the military community.

**Electronic supplementary material:**

The online version of this article (10.1186/s12889-018-5781-2) contains supplementary material, which is available to authorized users.

## Background

The U.S. Military maintains extensive records on the health of US Military personnel and their families via its healthcare system. However, information regarding health behaviors is not usually present in these records. Therefore, to systematically assess health behaviors among active duty personnel, the U.S. Department of Defense conducts a triennial Health Related Behaviors Survey (HRBS) of active duty military personnel [1], which assesses a range of self-reported health behaviors and risks, such as physical activity, diet, drug and alcohol use, posttraumatic stress, and doctor-informed medical conditions. The representative survey includes responses from participants in all branches of the Department of Defense [DoD] (Army, Navy, Marine Corps, and Air Force), as well as U.S. Coast Guard [USCG] members. The survey is voluntary and anonymous, and is used to guide the health-related policies in the Armed Forces. The HRBS provides data that are otherwise unavailable in this large subset of the U.S. population, approximately 1.3 million service members, since active duty military are excluded from national or state health-related surveys, such as the National Health and Nutrition Examination Survey (NHANES) or the Behavioral Risk Factor Surveillance System (BRFSS).

HRBS data have been used in a number of ways to study active duty and USCG personnel, for example, to identify whether they meet the *Healthy People 2010* objectives [2], to describe mental health patterns and associations [3–5], and assess changes over time in self-reported overweight/obesity [6, 7]. In depth analysis of demographic characteristics and health-related behaviors, and their association with self-reported health outcomes, may alter the manner in which military personnel are assessed and counseled for potential modifiable and non-modifiable demographic and behavioral risks. Furthermore, associations between health behaviors and disease risk factors may be different in military versus non-military populations due to differences in occupational demands and lifestyle characteristics. For example, deployment absences and frequent moves are sources of family stress that are more common in military than civilian families [8, 9]. Military personnel are required to meet standards of physical fitness and weight (or body composition) [10] and engage in regular physical training [11, 12]. Military personnel are part of a workforce with institutionalized standards and a unique warrior culture and ethos not present in the civilian workforce. [13] The unique occupational demands frequently involve high risk activities (e.g., combat), which are an inherent part of military service [14]. Also in contrast to civilians, and of specific relevance to the present research, military personnel and their dependents are eligible to receive healthcare at little or no cost [9].

Therefore, the aims of this study were to examine relationships between generally unmodifiable sociodemographic factors (e.g., age, sex, race/ethnicity) and self-reported health behaviors (e.g., exercise and diet), as well as relationships between these sociodemographic factors and self-reported, doctor-informed medical conditions (i.e., hypertension, dyslipidemia, and hyperglycemia) and overweight/obesity. We also examined the relationships between these self-reported health behaviors and the medical conditions.

## Methods

### Survey participants

The 2011 HRBS was conducted by ICF International under contract with the Office of the Assistant Secretary of Defense for Health Affairs, Tricare Management Activity (TMA; now Defense Health Agency), and the USCG [15]. Primary data collection was approved by the Office of the Assistant Secretary of Defense for Health Affairs/TRICARE Management Activity (OASD (HA)/TMA), Human Research Protection Office. Informed consent was required and obtained at the beginning of the HRBS, and responses were anonymous. For the present secondary data analyses, we obtained a de-identified data file from TMA through a data use agreement. Because the data were previously collected and de-identified, the protocol for the present study was deemed exempt (not human subject research) by both the U.S. Army Research Institute of Environmental Medicine (Natick, MA) and the Office of the Assistant Secretary of Defense for Health Affairs/TRICARE Management Activity (OASD (HA)/TMA), Human Research Protection Office.

The sampling strategy, survey administration, and questionnaire format are extensively described in the initial report of the 2011 HRBS [15]. In brief, a stratified random sample was drawn from all non-deployed active duty Army, Navy, Marine Corps, Air Force, and USCG personnel. A second stratified random sample was drawn within each service branch using a combination of sex and pay grade as stratification variables. The resulting group was randomly divided into a group to whom invitations to take the survey were sent, as well as two other groups to use in case of a low response rate. The USCG used a different sampling method, dividing its population into two groups, one of which was sampled using geographic clustering while the other was sampled using stratification on work setting, sex, and pay grade. The eligible sample of potential respondents was 168,664 (154,011 from the DoD services and 14,653 from the USCG) [15].

Invitations to participate in the survey and reminders to complete it were distributed via email. Participants were allowed to opt out of taking the survey. The survey was available for 5 months after the initial invitations were sent in August 2011 (October 2011 for USCG). Those who did not have listed email addresses were mailed physical invitations. The response rate was 22% from the DoD services, and 37% from the USCG, with a total of 39,877 usable, eligible responses [15]. Individuals who did not respond to questions on the variables of interest were excluded from the present analysis, for a final analytic sample size of 27,034. Characteristics of those included and excluded from the present analyses are provided in Additional file 1: Table S1.

All variables described below are derived from responses to the publicly available 2011 HRBS questionnaire [16].

### Sociodemographic variables

Sociodemographic variables included in the present analysis were service branch, sex, age, race/ethnicity, education, pay grade, marital status, and presence of minor children living with the respondent. Specifically, respondents were asked to report service branch (Army, Navy, Marine Corps, Air Force, Coast Guard), sex (male, female), age (< 20, 21–25, 26–35, 36–45, 46–65 years), education (up to high school or equivalent, some college without a degree, 2-year college degree, 4-year college degree, graduate education), race/ethnicity (non-Hispanic white, non-Hispanic black, Hispanic, and other/multiple race/ethnicity), pay grade (7 categories), current marital status (never married, married, separated/divorced/widowed), and number of children < 18 years living with the respondent at the duty station (0, ≥1 children).

### Health behavior variables

Health behaviors assessed included modifiable lifestyle factors: dietary intake, physical activity, cigarette smoking, alcohol intake, and sleep.

The 2011 HRBS questionnaire [16] asked about food intake during a given week, specifically the frequency of consumption of fruit (including “fresh, frozen, canned, or dried”), starchy vegetables (including “white potatoes, corn, peas”), vegetables (including “fresh, frozen, canned, cooked, or raw [not fried]”), whole grains (including “rye, whole-grain bread, brown or wild rice, whole-wheat pasta, oatmeal, etc.”), dairy (including “milk, yogurt, cheese, etc.”), lean protein (including “baked or broiled lean [low fat] meat, eggs, natural peanut butter, nuts, beans or legumes, tofu”), snack foods (including “potato chips, corn chips, pretzels”), sweets (including “chocolate, candy, cake, pie, breakfast bars, etc.”), sugary drinks (including “juice, regular soda, Kool-Aid, Yoo-hoo, sports drinks, etc.”), caffeinated drinks (including “coffee, tea, or energy drinks [Red Bull, Monster, 5-Hour Energy, Power Shots, etc.]”), and fried foods (including “French fries, fried chicken, donuts, etc.”). Response choices for each food were: rarely/never to ≥3 times per day. Each reported instance of eating a given food was counted as one serving of that food. For example, if a respondent reported they ate fruit twice per day, it was coded as two servings of fruit per day.

Respondents were asked about the frequency (daily to “not at all in the past 30 days”) and duration (≥60 min/day to “never in the past month”) of moderate (“exertion that raises heart rate and breathing, but you should be able to carry on a conversation comfortably during the activity”) and vigorous (“exertion that is high enough that you would find it difficult to carry on a conversation during the activity”) physical activity and strength training. Responses were recoded into minutes per week and summed for total physical activity; moderate and vigorous exercise minutes were also summed for total aerobic physical activity.

Cigarette smoking was classified into never-smoked, former smoker (abstinent for > 6 mo), or current smoker based on responses to questions on lifetime cigarette smoking, last smoking occasion, current smoking, and cigarettes per day.

Alcohol intake (drinks/day in last month) was quantified based on responses to questions on the average frequency and amount of alcohol consumed in the last month.

Sleep (hrs/d) was coded based on reported sleep hours in a typical 24-h period in the previous week. Reported typical sleep hours ≤2 or ≥ 16 h/d over the previous week were considered non-habitual (i.e., rare or due to extenuating circumstances such as periodic training exercise, illness, etc.).

### Doctor-informed medical condition and overweight/obesity variables

Medical conditions in the present analysis included overweight/obesity, and 4 doctor-informed health conditions (hypertension, hypercholesterolemia, low HDL cholesterol, hyperglycemia).

Respondents were asked to report their height (feet and inches) and their weight (pounds). Body mass index (BMI) was calculated as the weight (kg) divided by the square of the height (m). BMI values ≤12 or ≥ 50 kg/m^2^ were considered implausible.

Respondents were asked whether they had been told by a doctor or other health care professional that they had high blood pressure, high blood sugar, high cholesterol, low HDL cholesterol (“low amounts of good cholesterol”), or high triglycerides (“blood fat”). Response choices were “No”, “Yes, within the past 2 years,” or “Yes, more than 2 years ago”. Either yes response was coded as having the condition.

### Other covariates

In addition to the above, we included weight history based on whether a respondent reported having had to lose weight to enter service and if so, how much (5-lb categories ranging from 0 to ≥30 lbs). Respondents were also asked whether they were currently enrolled in mandatory weight control program (yes, no). A yes response was considered a marker of history of overweight/obesity, irrespective of current reported (calculated) BMI. We also assessed history of deployment since September 11, 2001 (yes, no).

### Statistical analysis

The original survey incorporated sample weights applied post-stratification to compensate for lower response rates and disparate selection probabilities across strata and enable the sample to resemble the active duty population as a whole. Separate weights were applied for USCG members based on the mode of sampling used with the goal to reflect the entire population of the USCG [15]. In the present analysis, we used a restricted sample with complete responses on questions/variables of interest (exclusions noted above). Because responses may be missing at random, or missing completely at random, for systematic reasons (e.g., survey length), or for biased reasons (e.g., undesirable response), and because such reasons are unknown, we did not impute responses of non-completers from completer data. However, to maintain consistency of the restricted sample with the respondent sample, and thus, with the active duty population, we adjusted the original sample weights by multiplying the original weights of respondents within each stratum (service, sex, pay grade) by the number of completers in each stratum. These adjusted sample weights were used in the present analyses.

For binary dependent variables, logistic regression was used to estimate odds ratios (OR) and 95% confidence intervals (CI) of the outcome; for continuous dependent variables, linear regression was used to estimate least square means and standard errors of the dependent variable in categories of the independent variables. Survey procedures available in SAS (v9.3, Cary, North Carolina) designed to deal with complex survey designs and sampling weights were used. Statistical significance was set at α = 0.05.

The present analyses evaluated the following associations: 1) demographic characteristics with health behaviors; 2) demographic characteristics with doctor-informed medical conditions and overweight/obesity, and 3) health behaviors with medical conditions. Models of demographic characteristics predicting either health behaviors or medical conditions included all demographic characteristics simultaneously, and additionally adjusted for enrollment in weight loss program, weight loss history, and deployment history. There were 3 models of health behavior associations with medical conditions: model 1 included all behaviors simultaneously; model 2 additionally adjusted for all demographic characteristics; model 3 additionally adjusted for enrollment in weight loss program, weight loss history, and deployment history. Results from all models can be found in the Additional file 1; for simplicity we present only the results of the fully adjusted model in the main text.

Given the large number of health behaviors evaluated, we also conducted secondary exploratory factor analysis to assess possible patterns within the 17 behaviors of diet, exercise, sleep, alcohol, smoking, that may predict medical conditions. Four factors (named “Healthy diet,” “Unhealthy diet,” “Exercise,” and “Bad habits”; Additional file 1: Table S2) were derived using a principal components approach, with varimax orthogonal rotation, retaining eigenvalues ≥1. Factors were named based on factor loadings >|0.20| and subsequently included in logistic regression models (models 1–3, as above, where behaviors were replaced with factors) assessing their associations with medical conditions.

## Results

Of the 27,034 respondents included in the analysis, 29, 17, 21, 19, and 14%, were in the Air Force, Army, Marines, Navy, and USCG, respectively (Table 1). The weighted mean (SEM) age of respondents was 29.9 (0.1) years; weighted percentages showed 14.7% were female, 69.8% identified as non-Hispanic white, 8.6% as non-Hispanic black, 25.5% had at least a high school education, 37.2% had completed some college, 58.9% were married, and 39.9% had one or more children residing with them. Just over 10% reported having to lose some weight to join a service, but only 3% reported being currently enrolled in a weight control program. A majority of respondents (61.3%) reported having been deployed at some point since September 11, 2001.

**Table 1:** Characteristics of 27,034 respondents to the 2011 Health Risk Behavior Survey, by service branch

	Air Force		Army		Marine Corps		Navy		Coast Guard		Total Sample	
Characteristics ^a^	N	Mean ± SEM or %	N	Mean ± SEM or %	N	Mean ± SEM or %	N	Mean ± SEM or %	N	Mean ± SEM or %	N	Mean ± SEM or %
Sociodemographic												
Age, y	7846	29.87 ± 0.09	4726	31.96 ± 0.15	5608	26.63 ± 0.10	5169	30.61 ± 0.13	3685	31.05 ± 0.13	27,034	29.86 ± 0.06
< 20 y	513	6.7	121	4.38	466	12.44	107	3.75	74	2.46	1281	6.35
20–≤25 y	2245	29.3	638	21.49	1619	43.47	911	28.34	761	24.36	6174	30.03
25–≤30 y	1707	23.9	862	22.96	1195	21.07	1051	24.25	910	26.64	5725	23.58
30–≤35 y	1169	15.8	851	17.94	875	10.54	977	17.14	759	20.87	4631	16.04
35–≤40 y	1220	13.6	947	16.54	841	7.69	970	14.39	572	13.29	4550	12.98
> 40 y	992	10.7	1307	16.70	612	4.78	1153	12.13	609	12.39	4673	11.02
Sex, female	2816	19.8	1647	14.3	1572	7.2	1936	16.6	556	13.17	8527	14.7
Education												
High school or equiv.	1096	15.9	531	19.99	1736	43.78	891	26.61	791	23.42	5045	25.5
Some college	2620	36.4	1435	37.34	2051	36.38	1553	35.62	1427	41.99	9086	37.2
2-year college	1608	19.0	656	12.51	420	5.84	757	11.88	391	10.37	3832	12.6
4-year college	862	9.5	819	15.30	816	9.18	852	12.08	481	12.46	3830	11.3
Beyond college	1660	19.2	1285	14.86	585	4.83	1116	13.81	595	11.76	5241	13.4
Race/ethnicity												
Non-Hispanic white	5736	74.3	3062	66.8	3699	67.0	3325	63.0	2861	77.4	18,683	69.8
Non-Hispanic black	686	7.6	719	12.0	542	8.1	680	11.4	135	3.4	2762	8.6
Hispanic	804	10.2	564	13.1	983	17.8	592	13.3	412	12.1	3355	13.1
Other/multiple	620	7.9	381	8.1	384	7.2	572	12.3	277	7.2	2234	8.5
Marital status												
Never married	2270	29.3	842	23.12	1559	39.75	1323	31.49	942	28.13	6936	30.7
Currently married	4669	60.4	3126	64.44	3388	51.27	3138	57.27	2365	62.21	16,686	58.9
Separated/divorced/ widowed	907	10.3	758	12.45	661	8.99	708	11.23	378	9.67	3412	10.5
Children, N	7846	0.81 ± 0.01	4726	0.85 ± 0.02	5608	0.60 ± 0.02	5169	0.75 ± 0.02	3685	0.79 ± 0.02	27,034	0.76 ± 0.01
None	4537	57.6	2496	57.31	3116	68.02	2769	60.14	2026	57.21	14,944	60.1
One or more	3309	42.40	2230	42.69	2492	31.98	2400	39.87	1659	42.79	12,090	39.9
Pay grade^b^												
E1-E4	2718	35.96	1205	47.48	1850	57.82	957	39.14	993	33.15	7723	42.7
E5-E6	2186	34.39	1084	24.88	1529	23.46	1572	34.70	1293	37.46	7664	30.9
E7-E9	1287	9.99	673	10.03	859	7.49	1069	8.89	481	10.79	4369	9.4
WO1-WO5	–	–	517	2.84	312	1.11	228	0.50	163	3.51	1220	1.3
O1-O3	1052	11.3	485	8.90	564	6.66	763	9.80	400	`	3259	9.3
O4-O10	603	8.34	762	5.87	494	3.45	580	6.97	355	6.14	2794	6.3
Enrolled in current weight control program	145	1.83	145	3.82	143	2.71	203	3.92	108	3.26	744	3.0
Weight loss to join service												
None	7076	89.87	4232	87.99	5000	87.03	4734	90.84	3387	91.34	24,429	89.3
< 5 lbs	70	0.80	39	0.92	56	0.74	35	0.58	26	0.66	226	0.8
5–9 lbs	186	2.22	143	3.03	156	2.38	102	1.93	74	2.22	661	2.3
10–14 lbs	201	2.75	119	2.50	139	2.93	123	2.21	59	1.67	641	2.5
15–19 lbs	84	1.13	51	1.30	73	1.89	54	1.35	45	1.41	307	1.4
20–29 lbs	108	1.45	48	1.23	73	1.76	59	1.27	38	1.05	326	1.4
≥ 30 lbs	121	1.78	94	3.04	111	3.26	62	1.82	56	1.65	444	2.3
Deployed since 9/2001	4384	57.25	3587	69.25	3820	57.96	3949	70.22	1980	52.58	17,720	61.26
Health behaviors												
Dietary intake (ser/wk)												
Fruit	7846	8.74 ± 0.08	4726	7.81 ± 0.12	5608	7.98 ± 0.11	5169	8.64 ± 0.12	3685	9.11 ± 0.11	27,034	8.45 ± 0.05
Starch		5.94 ± 0.06		6.11 ± 0.10		6.29 ± 0.10		6.04 ± 0.10		6.12 ± 0.09		6.09 ± 0.04
Vegetables		9.75 ± 0.08		9.12 ± 0.12		8.68 ± 0.11		9.54 ± 0.12		10.20 ± 0.11		9.44 ± 0.05
Whole grains		9.70 ± 0.08		9.24 ± 0.12		9.17 ± 0.11		9.24 ± 0.12		9.69 ± 0.11		9.42 ± 0.05
Dairy		9.61 ± 0.07		9.25 ± 0.13		8.89 ± 0.12		9.05 ± 0.12		9.97 ± 0.11		9.35 ± 0.05
Lean meat		9.81 ± 0.07		9.41 ± 0.13		9.48 ± 0.12		9.40 ± 0.12		10.12 ± 0.11		9.64 ± 0.05
Snacks		3.66 ± 0.05		3.68 ± 0.09		4.17 ± 0.09		3.89 ± 0.09		3.84 ± 0.08		3.84 ± 0.04
Sweets		3.61 ± 0.05		3.75 ± 0.09		3.80 ± 0.09		3.85 ± 0.09		3.66 ± 0.08		3.73 ± 0.04
Sugary drinks		4.68 ± 0.07		5.86 ± 0.14		6.22 ± 0.12		5.05 ± 0.12		4.39 ± 0.10		5.24 ± 0.05
Caffeinated drinks		7.38 ± 0.08		8.47 ± 0.14		7.66 ± 0.13		8.02 ± 0.13		9.25 ± 0.13		8.01 ± 0.05
Fried food		2.72 ± 0.04		2.85 ± 0.08		3.26 ± 0.08		3.04 ± 0.08		2.66 ± 0.06		2.90 ± 0.03
Exercise (min/wk)												
Moderate aerobic	7846	159.9 ± 1.5	4726	203.6 ± 2.7	5608	186.8 ± 2.5	5169	166.3 ± 2.4	3685	162.6 ± 2.3	27,034	174.7 ± 1.0
Vigorous aerobic		110.8 ± 1.2		130.5 ± 2.3		130.0 ± 2.2		104.1 ± 2.1		100.7 ± 1.9		115.6 ± 0.8
Strength training		97.8 ± 1.3		113.1 ± 2.3		124.6 ± 2.4		92.0 ± 2.2		93.5 ± 1.9		104.4 ± 0.9
Smoking												
Current	1221	16.63	945	26.88	1317	30.47	979	24.63	675	19.87	5137	23.24
Former	1337	17.00	882	18.28	1061	16.07	998	17.22	774	21.12	5052	17.64
Never	5288	66.38	2899	54.85	3230	53.45	3192	58.15	2236	59.02	16,845	59.11
Alcohol intake, drinks/d	7846	0.20 ± 0.01	4726	0.31 ± 0.01	5608	0.45 ± 0.02	5169	0.32 ± 0.01	3685	0.31 ± 0.01	27,034	0.31 ± 0.00
Non-drinker	1784	22.57	1072	23.25	1127	19.81	1021	20.47	527	14.76	5531	20.64
< 1/2 drink/d	4932	62.39	2564	54.06	2941	50.07	2967	55.46	2150	58.44	15,554	56.53
1/2–1 drink/d	978	12.69	872	16.28	1116	19.47	998	18.73	851	22.22	4815	17.17
1–< 2 drinks/d	107	1.64	152	4.29	253	6.08	124	3.11	109	3.20	745	3.52
2–< 4 drinks/d	34	0.52	49	1.66	139	3.69	47	1.74	36	1.06	305	1.68
≥ 4 drinks/d	11	0.19	17	0.47	32	0.88	12	0.47	12	0.32	84	0.45
Sleep, hrs/night	7846	6.58 ± 0.02	4726	6.05 ± 0.03	5608	6.14 ± 0.03	5169	6.24 ± 0.03	3685	6.55 ± 0.02	27,034	6.33 ± 0.01
< 5 h/night	503	6.45	616	15.10	674	13.51	536	12.65	186	4.77	2515	10.36
5–< 6 h/night	1158	14.49	960	21.71	1190	22.52	1031	20.06	551	14.63	4890	18.48
6–< 7 h/night	2433	31.56	1599	32.88	1835	31.87	1740	31.74	1278	34.35	8885	32.27
7–< 8 h/night	2196	28.01	967	18.71	1194	18.97	1186	21.51	1087	29.80	6630	23.54
8–< 9 h/night	1309	16.57	489	9.79	580	10.82	561	11.49	509	14.23	3448	12.92
9–< 10 h/night	176	2.11	58	1.03	67	1.05	57	1.20	44	1.27	402	1.42
≥ 10 h/night	71	0.81	37	0.76	68	1.26	58	1.34	30	0.94	264	1.01
Medical conditions												
High blood pressure	887	11.7	836	16.2	759	12.5	775	13.8	456	11.7	3713	13.0
High cholesterol	965	12.1	858	13.9	659	8.2	989	15.3	627	15.6	4098	12.7
High triglycerides	454	5.9	324	5.2	179	2.1	470	7.4	318	7.7	1745	5.5
Low HDL cholesterol	474	6.1	416	6.7	219	2.7	478	7.7	334	8.1	1921	6.1
High blood glucose	92	1.1	110	1.8	78	1.1	151	2.4	68	1.6	499	1.5
Body mass index, kg/m^2^	7846	25.74 ± 0.04	4726	26.57 ± 0.07	5608	25.36 ± 0.05	5169	26.42 ± 0.06	3685	26.33 ± 0.06	27,034	26.02 ± 0.03
Overweight/obese	4309	58.2	3128	67.8	3005	58.2	3358	66.0	2492	67.6	16,292	62.6
Total conditions, N	7846	0.37 ± 0.01	4726	0.44 ± 0.01	5608	0.27 ± 0.01	5169	0.47 ± 0.02	3685	0.45 ± 0.02	27,034	0.39 ± 0.01
≥ 1 condition(s)	1749	22.3	1528	27.1	1263	18.5	1607	26.5	1047	26.1	7194	23.7

^a^ Characteristics are weighted to representative populations

^b^ Pay grade E denotes Enlisted, W denotes Warrant Officer, and O denotes Officer

Weighted responses indicated low weekly intake of fried food, sweets, and snacks (~ 3–4 ser/wk), moderate intake of starches and sugary drinks (~ 5–6 ser/wk), and at least daily intake of fruit, vegetables, whole grains, dairy, lean meat, and caffeinated drinks (~ 8–10 ser/wk). Respondents reported 175, 116, and 104 min/wk. of moderate exercise, vigorous exercise, and strength training, respectively. Nearly 60% of the weighted sample reported never smoking. Average intake of alcohol in the prior month was a third of drink per day, with > 20% of the weighted sample reporting no alcohol intake. Average sleep duration was 6.3 h/night, with 32% reporting 6–7 h/night—the largest sleep category.

Nearly 24% of the sample reported having at least one medical condition of five possible doctor-informed conditions: 13.0% reported high blood pressure; 12.7% high cholesterol; 5.5% high triglycerides; 6.1% low HDL cholesterol; and 1.5% high blood glucose. Nearly 63% of the sample categorized themselves as overweight or obese, and mean BMI was 26 kg/m^2^.

### Associations of sociodemographic variables and health behaviors

In fully-adjusted models, several sociodemographic characteristics were associated with health behaviors. Exercise (both aerobic and strength training) duration was longer in younger age groups, males, those in the Army or Marine Corps, service members with higher educational attainment, separated/divorced/widowed, or individuals without children present (Table 2). Alcohol intake was highest in those age 21–25 y, and lower in higher age groups, was highest in those in the Marine Corps, males, in those with lower educational attainment, non-Hispanic whites, and was lowest among those currently married and those with children present. Average sleep time was longest in the youngest age category and in those in the Air Force or Coast Guard. Current smoking was less likely in the older age categories, among those in the Army or Marine Corps, in the currently or previously married, and was more likely in males, in those with higher educational attainment, and in non-Hispanic blacks.

**Table 2:** Associations between sociodemographic characteristics and health behaviors (i.e., exercise, alcohol, sleep, and smoking)^a^

		Mean ± SE					OR (95% CI)
Demographic Characteristic	Categories	Moderate exercise (min/wk)	Vigorous exercise (min/wk)	Strength training (min/wk)	Alcohol (drinks/d)	Sleep (hrs/night)	Current smoking
Age	≤20 yrs. (ref.)	171.9 ± 8.33	122.5 ± 8.28	104.3 ± 7.71	0.05 ± 0.03	6.5 ± 0.08	1
	21–25 yrs	155.1 ± 6.84	111.7 ± 7.24	**101.7 ± 6.33**	0.31 ± 0.02	6.3 ± 0.06	0.68 (0.57–0.80)
	26–35 yrs	150.7 ± 6.46	104.2 ± 7.00	88.8 ± 6.00	0.27 ± 0.02	6.2 ± 0.05	0.50 (0.41–0.60)
	36–45 yrs	137.4 ± 6.47	89.6 ± 7.02	70.4 ± 6.00	0.22 ± 0.02	6.1 ± 0.05	0.52 (0.42–0.64)
	46+ yrs	133.3 ± 7.41	81.3 ± 7.57	62.1 ± 6.51	0.20 ± 0.02	6.1 ± 0.06	0.46 (0.36–0.59)
Service	Army (ref.)	178.0 ± 6.78	119.1 ± 7.24	96.6 ± 6.28	0.22 ± 0.02	6.0 ± 0.06	1
	Air Force	135.0 ± 6.45	97.6 ± 7.00	81.9 ± 5.99	0.11 ± 0.02	6.5 ± 0.05	1.66 (1.50–1.83)
	Coast Guard	137.4 ± 6.79	88.3 ± 7.20	77.6 ± 6.23	**0.20 ± 0.02**	6.5 ± 0.06	1.46 (1.30–1.63)
	Marine Corps	155.5 ± 6.74	112.1 ± 7.20	**97.0 ± 6.28**	0.32 ± 0.02	**6.0 ± 0.06**	**1.04 (0.92–1.17)**
	Navy	142.5 ± 6.70	92.2 ± 7.14	74.3 ± 6.19	**0.22 ± 0.02**	6.2 ± 0.06	1.18 (1.05–1.32)
Sex	Female (ref.)	141.6 ± 6.45	89.6 ± 6.99	66.1 ± 5.96	0.14 ± 0.02	6.3 ± 0.05	1
	Male	157.7 ± 6.42	114.1 ± 6.98	104.8 ± 5.98	0.29 ± 0.02	6.2 ± 0.05	1.24 (1.16–1.33)
Education	High school or equiv. (ref.)	139.6 ± 6.82	91.5 ± 7.26	79.3 ± 6.33	0.27 ± 0.02	6.2 ± 0.06	1
	Some college	149.4 ± 6.62	99.9 ± 7.12	84.7 ± 6.12	0.22 ± 0.02	**6.1 ± 0.06**	1.26 (1.15–1.38)
	2-year college	**145.6 ± 6.88**	101.4 ± 7.31	87.4 ± 6.36	0.20 ± 0.02	**6.2 ± 0.06**	1.65 (1.47–1.85)
	4-year college	160.0 ± 6.87	106.5 ± 7.26	89.8 ± 6.31	0.18 ± 0.02	6.3 ± 0.06	2.73 (2.38–3.13)
	Beyond college	153.8 ± 6.93	110.0 ± 7.32	**86.0 ± 6.34**	0.18 ± 0.02	6.4 ± 0.06	3.28 (2.79–3.84)
Race/ethnicity	Non-Hispanic white (ref.)	155.0 ± 6.36	101.4 ± 6.93	74.8 ± 5.91	0.27 ± 0.02	6.3 ± 0.05	1
	Non-Hispanic black	144.3 ± 6.89	**95.7 ± 7.34**	90.0 ± 6.39	0.16 ± 0.02	**6.2 ± 0.06**	3.57 (3.11–4.09)
	Hispanic	**151.6 ± 6.86**	108.8 ± 7.30	91.4 ± 6.39	0.22 ± 0.02	**6.3 ± 0.06**	1.73 (1.56–1.92)
	Other/multiple	**147.9 ± 7.12**	**101.5 ± 7.47**	85.6 ± 6.58	0.18 ± 0.02	6.1 ± 0.06	1.17 (1.04–1.32)
Marital status	Never married (ref.)	144.6 ± 6.62	101.8 ± 7.11	83.4 ± 6.14	0.23 ± 0.02	6.3 ± 0.06	1
	Currently married	**150.3 ± 6.36**	**99.4 ± 6.94**	**80.6 ± 5.93**	0.15 ± 0.02	**6.4 ± 0.05**	0.85 (0.77–0.93)
	Separated/divorced/ widowed	154.1 ± 6.98	**104.4 ± 7.32**	92.4 ± 6.44	**0.25 ± 0.02**	6.0 ± 0.06	0.59 (0.52–0.67)
Children	0 children (ref.)	154.0 ± 6.37	105.2 ± 6.94	89.3 ± 5.93	0.24 ± 0.02	6.3 ± 0.05	1
	1+ children	145.3 ± 6.56	98.5 ± 7.07	81.5 ± 6.08	0.18 ± 0.02	6.2 ± 0.05	0.89 (0.83–0.96)
Pay grade	E1-E4 (ref.)	176.1 ± 2.82	**107.1 ± 2.36**	99.5 ± 2.40	0.16 ± 0.01	6.3 ± 0.03	1
	E5-E6	167.7 ± 2.73	**108.4 ± 2.30**	**97.9 ± 2.33**	**0.18 ± 0.01**	6.2 ± 0.03	0.82 (0.75–0.91)
	E7-E9	165.3 ± 3.14	**111.0 ± 2.61**	**97.3 ± 2.66**	0.24 ± 0.01	**6.3 ± 0.03**	**0.93 (0.82–1.06)**
	WO1-WO5	161.5 ± 5.60	**102.3 ± 4.35**	87.9 ± 4.30	0.24 ± 0.02	6.5 ± 0.05	**1.12 (0.91–1.37)**
	O1-O3	150.3 ± 3.64	**102.7 ± 3.06**	87.0 ± 3.10	0.22 ± 0.01	6.5 ± 0.03	1.72 (1.48–2.00)
	O4-O10	143.3 ± 4.28	**101.9 ± 3.57**	82.3 ± 3.42	0.30 ± 0.01	6.4 ± 0.04	2.24 (1.84–2.73)

^a^ Reference categories are indicated for tests of differences between means or for odds ratios. All differences from the reference category are statistically significant (*P* < 0.05), except for means or odds ratios in bold. Linear regressions to estimate least square means, and logistic regressions to estimate odds ratios were adjusted for sample weights. Multivariate analyses included all demographic characteristics simultaneously

Dietary intake of all food groups except vegetables was highest in the youngest age category, and was lower in progressively older age categories (Table 3). Differences in food intake (e.g., starch and fried food) between service branches were small (< 2 ser/wk) or statistically the same across all sociodemographic characteristics. Females reported higher intake than males of fruit, vegetables, and dairy, and lower intake of sugary drinks and fried foods. Those with higher education reported higher intakes of fruit, vegetables, whole grains, and dairy than those with lower education. Those who reported being currently married or having children present reported higher intake of fruit, starches, vegetables, whole grains, and dairy relative to their not married or childless counterparts.

**Table 3:** Means of dietary intake by sociodemographic characteristics^a^

		Servings/week (mean ± SE)								
Characteristic	Categories	Fruit	Starch	Vegetables	Whole grains	Dairy	Sweets	Sugary drinks	Caffeinated drinks	Fried food
Age	≤20 yrs. (ref.)	9.5 ± 0.41	6.3 ± 0.35	**10.7 ± 0.40**	10.9 ± 0.46	10.5 ± 0.67	4.7 ± 0.26	4.9 ± 0.36	5.7 ± 0.33	3.3 ± 0.23
	21–25 yrs	9.0 ± 0.34	5.7 ± 0.29	**10.6 ± 0.33**	10.3 ± 0.40	10.0 ± 0.63	4.0 ± 0.18	4.2 ± 0.26	**6.0 ± 0.24**	2.7 ± 0.14
	26–35 yrs	8.9 ± 0.32	5.5 ± 0.27	**10.9 ± 0.31**	9.8 ± 0.39	9.3 ± 0.62	3.8 ± 0.16	3.7 ± 0.24	7.0 ± 0.21	2.4 ± 0.12
	36–45 yrs	8.3 ± 0.33	5.0 ± 0.27	**10.4 ± 0.32**	8.8 ± 0.39	8.2 ± 0.62	3.3 ± 0.16	3.3 ± 0.24	8.1 ± 0.21	2.1 ± 0.12
	46+ yrs	8.3 ± 0.37	4.4 ± 0.30	**10.4 ± 0.36**	8.2 ± 0.42	7.7 ± 0.64	3.1 ± 0.20	2.8 ± 0.28	9.0 ± 0.31	1.8 ± 0.14
Service	Army (ref.)	8.1 ± 0.34	5.5 ± 0.28	10.2 ± 0.33	9.5 ± 0.40	9.2 ± 0.63	3.9 ± 0.18	4.6 ± 0.26	7.4 ± 0.23	2.5 ± 0.14
	Air Force	8.8 ± 0.33	**5.2 ± 0.27**	10.6 ± 0.31	**9.7 ± 0.39**	**9.2 ± 0.62**	3.6 ± 0.16	3.5 ± 0.24	6.3 ± 0.21	**2.4 ± 0.12**
	Coast Guard	9.5 ± 0.34	**5.4 ± 0.28**	11.2 ± 0.33	9.9 ± 0.40	9.7 ± 0.63	**3.8 ± 0.17**	3.1 ± 0.25	**7.7 ± 0.24**	**2.4 ± 0.13**
	Marine Corps	8.6 ± 0.34	**5.4 ± 0.28**	**10.2 ± 0.33**	**9.3 ± 0.40**	8.6 ± 0.63	**3.7 ± 0.17**	4.0 ± 0.26	**7.2 ± 0.23**	**2.5 ± 0.14**
	Navy	9.0 ± 0.34	**5.4 ± 0.28**	10.7 ± 0.33	**9.5 ± 0.40**	**9.0 ± 0.63**	**3.9 ± 0.17**	3.7 ± 0.25	**7.1 ± 0.23**	**2.6 ± 0.14**
Sex	Female (ref.)	9.3 ± 0.33	4.8 ± 0.27	11.0 ± 0.31	9.4 ± 0.39	9.3 ± 0.62	3.8 ± 0.16	2.9 ± 0.24	6.7 ± 0.21	2.0 ± 0.12
	Male	8.3 ± 0.32	6.0 ± 0.27	10.2 ± 0.31	9.8 ± 0.39	8.9 ± 0.62	**3.7 ± 0.16**	4.6 ± 0.24	7.6 ± 0.20	3.0 ± 0.12
Education	High school or equiv. (ref.)	8.0 ± 0.34	5.2 ± 0.29	9.8 ± 0.33	8.7 ± 0.40	8.8 ± 0.63	3.9 ± 0.18	5.0 ± 0.26	7.6 ± 0.24	2.8 ± 0.14
	Some college	8.5 ± 0.33	**5.4 ± 0.28**	10.4 ± 0.32	9.3 ± 0.39	9.1 ± 0.62	**3.5 ± 0.16**	4.0 ± 0.24	7.2 ± 0.22	2.6 ± 0.13
	2-year college	8.9 ± 0.35	5.6 ± 0.29	10.6 ± 0.34	9.7 ± 0.40	**9.1 ± 0.63**	3.7 ± 0.18	3.4 ± 0.26	**7.3 ± 0.25**	2.4 ± 0.14
	4-year college	9.2 ± 0.34	**5.3 ± 0.29**	10.8 ± 0.33	10.0 ± 0.40	9.3 ± 0.63	4.0 ± 0.18	3.4 ± 0.26	6.8 ± 0.24	2.4 ± 0.14
	Beyond college	9.5 ± 0.35	**5.4 ± 0.29**	11.4 ± 0.34	10.3 ± 0.40	9.4 ± 0.63	3.9 ± 0.18	3.1 ± 0.26	7.0 ± 0.25	2.2 ± 0.14
Race/ethnicity	Non-Hispanic white (ref.)	9.0 ± 0.32	5.6 ± 0.27	11.3 ± 0.31	10.2 ± 0.38	10.4 ± 0.62	3.7 ± 0.15	3.7 ± 0.23	9.3 ± 0.20	2.2 ± 0.12
	Non-Hispanic black	8.5 ± 0.35	5.3 ± 0.29	10.0 ± 0.34	8.9 ± 0.41	8.1 ± 0.63	4.1 ± 0.19	4.8 ± 0.27	4.6 ± 0.23	3.0 ± 0.16
	Hispanic	8.6 ± 0.34	5.0 ± 0.29	10.0 ± 0.33	9.2 ± 0.40	9.1 ± 0.63	3.4 ± 0.18	3.2 ± 0.26	6.8 ± 0.23	**2.1 ± 0.14**
	Other/multiple	**9.1 ± 0.36**	**5.7 ± 0.30**	**11.2 ± 0.35**	**10.1 ± 0.41**	8.9 ± 0.64	**3.8 ± 0.19**	3.4 ± 0.27	7.9 ± 0.26	2.5 ± 0.15
Marital status	Never married (ref.)	8.6 ± 0.33	5.4 ± 0.28	10.3 ± 0.32	9.5 ± 0.39	8.8 ± 0.62	3.9 ± 0.17	3.8 ± 0.25	6.9 ± 0.22	2.7 ± 0.13
	Currently married	9.0 ± 0.32	5.5 ± 0.27	11.0 ± 0.31	10.0 ± 0.38	9.6 ± 0.62	**3.8 ± 0.15**	**3.6 ± 0.23**	**7.0 ± 0.20**	2.2 ± 0.12
	Separated/divorced/ widowed	**8.8 ± 0.35**	5.2 ± 0.29	**10.4 ± 0.34**	**9.4 ± 0.40**	**9.0 ± 0.63**	**3.7 ± 0.18**	**3.9 ± 0.27**	7.5 ± 0.25	**2.5 ± 0.15**
Children	0 children (ref.)	8.5 ± 0.32	5.2 ± 0.27	10.3 ± 0.31	9.4 ± 0.38	8.7 ± 0.62	3.8 ± 0.15	3.8 ± 0.23	7.0 ± 0.20	2.6 ± 0.12
	1+ children	9.1 ± 0.33	5.6 ± 0.28	10.9 ± 0.32	9.8 ± 0.39	9.6 ± 0.62	**3.8 ± 0.16**	**3.7 ± 0.24**	7.3 ± 0.22	2.4 ± 0.13
Pay grade	E1-E4 (ref.)	8.9 ± 0.14	5.7 ± 0.12	**9.3 ± 0.13**	9.0 ± 0.13	8.7 ± 0.13	3.7 ± 0.10	4.8 ± 0.13	6.0 ± 0.15	2.8 ± 0.08
	E5-E6	**8.7 ± 0.13**	**5.5 ± 0.11**	**9.4 ± 0.13**	8.7 ± 0.13	**8.5 ± 0.13**	**3.8 ± 0.10**	**4.6 ± 0.13**	7.2 ± 0.14	2.6 ± 0.08
	E7-E9	8.5 ± 0.15	5.2 ± 0.12	**9.3 ± 0.15**	8.4 ± 0.15	8.2 ± 0.15	**3.8 ± 0.11**	4.0 ± 0.15	8.1 ± 0.18	2.6 ± 0.09
	WO1-WO5	**8.7 ± 0.27**	5.1 ± 0.19	**8.9 ± 0.25**	8.1 ± 0.25	**8.2 ± 0.25**	**3.9 ± 0.18**	3.5 ± 0.21	8.5 ± 0.31	**2.7 ± 0.14**
	O1-O3	**9.3 ± 0.18**	5.1 ± 0.14	**9.5 ± 0.18**	**9.0 ± 0.18**	**9.0 ± 0.17**	4.1 ± 0.13	3.8 ± 0.16	7.2 ± 0.20	2.5 ± 0.10
	O4-O10	**9.3 ± 0.21**	5.2 ± 0.16	**9.2 ± 0.21**	**8.6 ± 0.21**	**8.8 ± 0.20**	4.7 ± 0.15	3.6 ± 0.18	8.4 ± 0.25	**2.7 ± 0.11**

^a^ Reference categories are indicated for tests of differences between means. All differences from the reference category are statistically significant (*P* < 0.05), except for means in bold. Linear regressions to estimate least square means were adjusted for sample weights. Multivariate analyses included all demographic characteristics simultaneously

### Demographic variables associated with medical conditions

In the fully-adjusted models, specific sociodemographic characteristics were associated with doctor-informed medical conditions and overweight/obesity (Table 4; Additional file 1: Table S3). Odds of all conditions were higher amongst older age compared to the youngest age category, and in males compared to females, except for high blood glucose for which females had higher odds than males.

**Table 4:** Odds ratios (95%CI) of medical conditions by sociodemographic characteristics ^a^

Characteristic	Categories	High blood pressure	High cholesterol	High triglycerides	Low HDL cholesterol	High blood glucose	Overweight/ obesity
Cases (N)		3713	4098	1745	1921	499	16,292
Age	21–25 yrs	**1.69 (1.13–2.53)**	1.27 (0.67–2.38)	1.25 (0.41–3.83)	1.21 (0.45–3.25)	1.13 (0.34–3.79)	**1.54 (1.28–1.84)**
(vs. ≤20 yrs)	26–35 yrs	**2.30 (1.51–3.50)**	**2.94 (1.57–5.50)**	2.59 (0.87–7.74)	**2.79 (1.05–7.38)**	1.34 (0.40–4.54)	**2.21 (1.80–2.70)**
	36–45 yrs	**3.77 (2.43–5.83)**	**8.12 (4.28–15.38)**	**7.94 (2.63–23.93)**	**6.86 (2.57–18.32)**	**3.54 (1.04–12.06)**	**3.82 (3.05–4.78)**
	46+ yrs	**6.33 (4.01–10.01)**	**12.54 (6.54–24.06)**	**11.81 (3.88–36.00)**	**11.80 (4.37–31.88)**	**10.06 (2.85–35.52)**	**4.19 (3.22–5.45)**
Service Branch	Air Force	**0.80 (0.70–0.91)**	0.98 (0.86–1.11)	**1.26 (1.05–1.52)**	0.97 (0.82–1.15)	0.73 (0.52–1.02)	**0.79 (0.71–0.88)**
(vs. Army)	Coast Guard	**0.72 (0.62–0.85)**	**1.24 (1.07–1.44)**	**1.59 (1.29–1.95)**	**1.27 (1.04–1.54)**	1.01 (0.68–1.50)	1.05 (0.93–1.19)
	Marine Corps	0.98 (0.84–1.15)	0.95 (0.81–1.11)	**0.68 (0.52–0.88)**	**0.66 (0.53–0.83)**	1.01 (0.69–1.50)	**0.78 (0.69–0.88)**
	Navy	**0.85 (0.73–0.99)**	**1.24 (1.07–1.43)**	**1.57 (1.28–1.91)**	**1.25 (1.04–1.51)**	**1.45 (1.05–2.02)**	1.04 (0.91–1.18)
Sex (vs. male)	Female	**0.54 (0.49–0.60)**	**0.65 (0.58–0.72)**	**0.56 (0.47–0.66)**	**0.47 (0.40–0.55)**	**1.35 (1.03–1.77)**	**0.29 (0.27–0.31)**
Education	Some college	0.99 (0.87–1.14)	1.15 (0.98–1.34)	1.18 (0.94–1.48)	**1.25 (1.00–1.56)**	0.95 (0.66–1.36)	1.03 (0.93–1.14)
(vs. high school or equiv.)	2-year college	**0.84 (0.71–0.99)**	1.13 (0.95–1.34)	**1.33 (1.03–1.70)**	**1.50 (1.18–1.91)**	1.01 (0.67–1.51)	**0.88 (0.77–1.00)**
	4-year college	**0.84 (0.69–1.00)**	1.19 (0.98–1.43)	**1.59 (1.22–2.08)**	**1.52 (1.18–1.96)**	0.62 (0.40–0.98)	0.94 (0.81–1.09)
	Beyond college	0.89 (0.72–1.08)	**1.39 (1.13–1.71)**	**1.73 (1.30–2.30)**	**1.92 (1.45–2.56)**	1.11 (0.67–1.82)	**0.83 (0.70–0.98)**
Race/ethnicity	Hispanic	0.89 (0.77–1.03)	1.06 (0.91–1.22)	1.04 (0.84–1.27)	0.90 (0.73–1.10)	**1.39 (1.00–1.95)**	**1.43 (1.28–1.59)**
(vs. non-Hispanic white)	Non-Hispanic black	**1.68 (1.46–1.93)**	1.11 (0.96–1.30)	**0.58 (0.44–0.75)**	**0.78 (0.62–0.98)**	1.42 (0.99–2.05)	**1.36 (1.20–1.55)**
	Other/multiple	**1.43 (1.23–1.67)**	**1.44 (1.23–1.67)**	**1.37 (1.12–1.67)**	1.20 (0.98–1.47)	**1.90 (1.36–2.66)**	0.91 (0.80–1.03)
Marital status	Currently married	1.12 (0.97–1.31)	**1.26 (1.08–1.48)**	1.03 (0.81–1.30)	0.96 (0.76–1.20)	1.11 (0.72–1.72)	**1.25 (1.14–1.38)**
(vs. never married)	Separated/divorced/ widowed	**1.31 (1.10–1.57)**	1.18 (0.98–1.42)	0.93 (0.71–1.23)	0.96 (0.74–1.25)	1.42 (0.89–2.26)	1.07 (0.94–1.21)
Children (vs. none)	1+ children	1.06 (0.96–1.18)	1.08 (0.98–1.20)	**1.24 (1.08–1.43)**	**1.32 (1.16–1.52)**	**1.64 (1.27–2.11)**	**1.23 (1.13–1.34)**
Pay grade	E5-E6	**1.57 (1.34–1.85)**	**1.90 (1.58–2.28)**	**2.10 (1.60–2.76)**	**1.86 (1.41–2.46)**	**1.63 (1.09–2.45)**	**1.54 (1.39–1.72)**
(vs. E1-E4)	E7-E9	**1.68 (1.39–2.03)**	**2.07 (1.68–2.55)**	**2.22 (1.64–3.00)**	**2.13 (1.57–2.89)**	1.41 (0.90–2.21)	**1.73 (1.50–2.01)**
	WO1-WO5	**1.41 (1.10–1.82)**	**2.28 (1.76–2.95)**	**2.23 (1.53–3.24)**	**2.25 (1.55–3.26)**	1.00 (0.55–1.81)	**1.87 (1.49–2.35)**
	O1-O3	1.12 (0.90–1.39)	**1.41 (1.13–1.77)**	**1.42 (1.03–1.97)**	1.34 (0.96–1.86)	1.05 (0.61–1.80)	**1.18 (1.01–1.37)**
	O4-O10	1.15 (0.90–1.47)	**1.63 (1.27–2.09)**	**1.49 (1.05–2.12)**	**1.58 (1.11–2.27)**	0.86 (0.48–1.54)	**1.27 (1.04–1.55)**

^a^ Logistic regressions were weighted by sample weights. Fully-adjusted models included all sociodemographic characteristics simultaneously, current enrollment in a weight-loss program, history of weight loss, and history of deployment. In bold text are odds ratios that are significantly different from the reference category. For simpler (less adjusted) models, see Additional file 1: Table S3

There were no discernible patterns in reported doctor-informed medical conditions and overweight/obesity between the service branches. However, those in the Navy had markedly higher odds of high blood glucose (~ 45% higher odds compared with the Army) and those in the Coast Guard or Navy had markedly higher odds of high triglycerides (~ 54–59% higher odds compared with the Army) (Table 4).

Higher educational attainment tended to be associated with incrementally higher odds of high cholesterol, low HDL cholesterol, high triglycerides, and with lower odds of overweight/obesity, associations which were not accounted for by simultaneously adjusting for age. For example, relative to those with a high school education or equivalent, those with some college, 2 years of college, 4 years of college, and those with education beyond college, had 1.25, 1.50, 1.52, and 1.92 times the odds, respectively, of having low HDL cholesterol (Table 4).

Compared to non-Hispanic whites, non-Hispanic blacks had 68% higher odds of high blood pressure, 36% higher odds of overweight/obesity, 42% lower odds of high triglycerides, and 22% lower odds of HDL. Hispanic personnel had 39% higher odds of high blood glucose, and 43% higher odds of overweight/obesity relative to non-Hispanic whites (Table 4).

Currently married respondents, compared to those who were never married, had significantly higher odds of high cholesterol and overweight/obesity, while those who were separated/divorced/widowed had significantly higher odds of high blood pressure. Having children present in the home was also associated with having a medical condition: compared to those without children living with them, those with children had 24, 32, 64, and 23% higher odds of high triglycerides, low HDL cholesterol, high blood glucose, and overweight/obesity, respectively (Table 4).

### Behaviors associated doctor-informed medical conditions and overweight/obesity

**Fig. 1:**
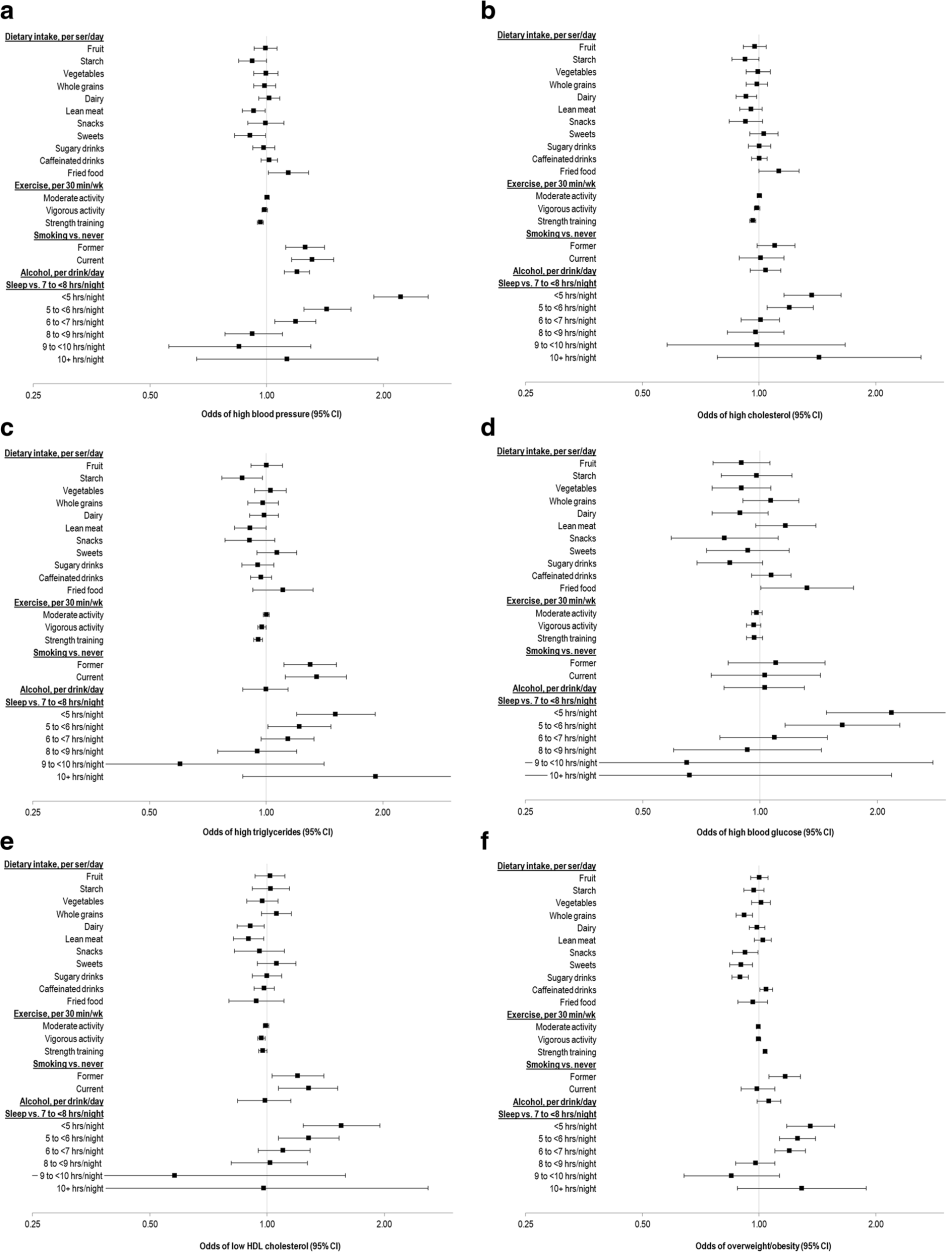
Odds of doctor-informed medical conditions according to health behaviors. Forest plot of odds of having self-reported high blood pressure (**a**), cholesterol (**b**), triglycerides (**c**), blood glucose (**d**), low high-density lipoprotein [HDL] cholesterol (**e**), or being overweight/obese (**f**) by health behaviors. Models were adjusted for all behaviors simultaneously, sociodemographic characteristics (age, sex, service branch, education, race/ethnicity, marital status, children living with the respondent, and pay grade), current enrollment in a weight-loss program, history of weight loss, and history of deployment. Odds ratios are given by the enclosed squares; 95% confidence intervals are given by the bars. For related data, see Additional file 1: Table S4

Of the 17 health behaviors assessed, the health behavior most consistently associated with every doctor-informed medical condition and overweight/obesity was shorter duration of sleep. In fully adjusted models, compared to sleeping 7–8 h/night, sleeping < 5 h/night was associated with 1.36 (95% CI 1.18–1.57, for overweight/obesity) to up to 2.22 (95% CI 1.89–2.61, for hypertension) times the odds of having a medical condition (Fig. 1a–f; Additional file 1: Table S4). Odds of most conditions also tended to be higher at sleep durations < 6 h/night, relative to 7–8 h/night. Sleep durations of 8 h/night or longer were not statistically different from 7 to 8 h/night.

In addition, in fully adjusted models, strength training was associated with 2–5% lower odds of lipid-related conditions (i.e., high cholesterol, high triglycerides, and low HDL) per 30 min/wk. increment, as well as high blood pressure, but slightly higher odds of overweight/obesity (4% per 30 min/wk. increment). Neither vigorous nor moderate exercise was consistently associated with medical conditions. Compared with never-smoking, current smoking, in fully adjusted models, was associated with higher odds of high blood pressure, low HDL cholesterol, and high triglycerides; only former smoking was associated with higher odds of overweight/obesity. Alcohol intake was also associated with higher odds of high blood pressure and blood glucose (Fig. 1a–f; Additional file 1: Table S4).

Among the 11 dietary factors, across models, lean meats and sweets were associated with lower odds of high blood pressure, dairy with lower odds of high cholesterol, starch with lower odds of high triglycerides, and dairy and lean meat with lower odds of low HDL cholesterol. Intake of whole grains, snacks, sweets, and sugary drinks were associated with significantly lower odds of overweight/obesity, while caffeinated drinks were associated with higher odds (Fig. 1a–f; Additional file 1: Table S4).

### Factor analyses of behaviors and risks

Exploratory factor analyses conducted found that 4 factors explained 9% of the variance in the original behavioral variables (Additional file 1: Table S2). They were characterized as “Healthy diet” (based on high loadings of fruit, starch, vegetable, whole grain, dairy, and lean meat intake), “Unhealthy diet” (based on high loadings of starch, snacks, sweets, sugary drinks, fried food, and caffeinated drinks), “Exercise” (based on high loadings of moderate and vigorous aerobic exercise and strength training), and “Bad habits” (based on high loadings of alcohol, smoking, and caffeinated drinks, and inverse loading of sleep). The factors were associated with medical conditions in generally expected ways: higher “Healthy diet” was consistently inversely associated with odds of high blood pressure, high cholesterol, high triglycerides, low HDL cholesterol, and overweight/obesity (Table 5). Higher “Unhealthy diet” was only consistently associated with *lower* odds of overweight/obesity. “Exercise” was consistently associated with lower odds of high cholesterol, high triglycerides, high blood glucose, and low HDL cholesterol, and was associated with *higher* odds overweight/obesity. Finally, “Bad habits” was strongly associated with higher odds of high blood pressure, high cholesterol, high triglycerides, high blood glucose, low HDL cholesterol, and overweight/obesity.

**Table 5:** Odds ratios (95% CI) of medical conditions by derived behavioral factors ^a^

Factor	Name	Model 1	Model 2	Model 3
		Odds of high blood pressure (N cases = 3713)		
1	“Healthy diet”	**0.86 (0.81–0.90)**	**0.88 (0.83–0.93)**	**0.87 (0.83–0.93)**
2	“Unhealthy diet”	**0.89 (0.83–0.94)**	0.96 (0.90–1.02)	0.97 (0.91–1.03)
3	“Exercise”	**0.89 (0.84–0.94)**	0.95 (0.90–1.01)	0.95 (0.89–1.01)
4	“Bad habits”	**1.40 (1.34–1.48)**	**1.41 (1.33–1.49)**	**1.39 (1.31–1.48)**
		Odds of high cholesterol (N cases = 4098)		
1	“Healthy diet”	**0.87 (0.82–0.91)**	**0.83 (0.79–0.88)**	**0.83 (0.79–0.88)**
2	“Unhealthy diet”	**0.83 (0.78–0.88)**	1.00 (0.93–1.06)	1.00 (0.94–1.07)
3	“Exercise”	**0.70 (0.66–0.74)**	**0.85 (0.80–0.90)**	**0.85 (0.80–0.90)**
4	“Bad habits”	**1.08 (1.03–1.13)**	**1.10 (1.04–1.17)**	1.10 (1.03–1.17)
		Odds of high triglycerides (N cases = 1745)		
1	“Healthy diet”	**0.89 (0.83–0.96)**	**0.83 (0.77–0.90)**	**0.83 (0.77–0.90)**
2	“Unhealthy diet”	**0.77 (0.71–0.84)**	0.93 (0.85–1.02)	0.94 (0.85–1.03)
3	“Exercise”	**0.61 (0.56–0.67)**	**0.79 (0.72–0.86)**	**0.78 (0.71–0.85)**
4	“Bad habits”	**1.11 (1.04–1.18)**	**1.16 (1.07–1.26)**	**1.15 (1.06–1.26)**
		High blood glucose (N cases = 499)		
1	“Healthy diet”	**0.85 (0.73–0.99)**	0.87 (0.76–1.01)	0.87 (0.75–1.00)
2	“Unhealthy diet”	**0.72 (0.61–0.86)**	0.85 (0.72–1.00)	0.87 (0.74–1.03)
3	“Exercise”	**0.64 (0.52–0.77)**	**0.75 (0.62–0.91)**	**0.74 (0.61–0.90)**
4	“Bad habits”	**1.21 (1.08–1.36)**	**1.25 (1.09–1.44)**	**1.25 (1.08–1.44)**
		Low HDL cholesterol (N cases = 1921)		
1	“Healthy diet”	**0.91 (0.85–0.98)**	**0.86 (0.80–0.93)**	**0.86 (0.80–0.93)**
2	“Unhealthy diet”	**0.81 (0.75–0.88)**	0.97 (0.89–1.06)	0.98 (0.90–1.07)
3	“Exercise”	**0.63 (0.58–0.69)**	**0.79 (0.72–0.87)**	**0.79 (0.72–0.86)**
4	“Bad habits”	**1.11 (1.04–1.19)**	**1.16 (1.07–1.27)**	**1.16 (1.06–1.26)**
		Odds of overweight/obesity (N cases = 16,292)		
1	“Healthy diet”	**0.94 (0.91–0.98)**	**0.94 (0.91–0.99)**	**0.95 (0.91–0.99)**
2	“Unhealthy diet”	**0.75 (0.72–0.78)**	**0.78 (0.74–0.81)**	**0.80 (0.76–0.83)**
3	“Exercise”	**1.05 (1.01–1.09)**	**1.11 (1.06–1.16)**	**1.12 (1.07–1.18)**
4	“Bad habits”	**1.20 (1.15–1.25)**	**1.10 (1.05–1.16)**	**1.09 (1.04–1.15)**

^a^ Logistic regressions were weighted by sample weights and can be interpreted as odds of the outcome per unit increase in the factor score. Model 1 was adjusted for all factors simultaneously. Model 2 was additionally adjusted for all sociodemographic characteristics (see Table 1). Model 3 was additionally adjusted for current enrollment in a weight-loss program, history of weight loss, and history of deployment. For additional details on the derived factors, see Additional file 1: Table S2. In bold text are odds ratios that are significantly different from the reference category

## Discussion

We used a large anonymous self-reported survey of the Armed Forces to assess relationships between sociodemographic characteristics, health behaviors, and doctor-informed medical conditions and overweight/obesity. When we examined individual health behaviors and medical conditions, by far the most consistent and largest relationship was between low sleep duration and higher odds of every medical condition. Otherwise, other behaviors, including dietary and exercise behaviors, were inconsistently associated with medical conditions. Age, sex, marital status, and presence of children appeared to be the only relatively consistent sociodemographic factors related to health behaviors. Older individuals, females, and those residing with children tended to exercise less, but eat healthier foods and not smoke. Older individuals also slept less, as did those with children present. Those who were married appeared to have consumed a better diet, exercised more, and slept more than their non-married counterparts. In terms of medical conditions, older age and female sex appeared to be the only consistent drivers of higher and lower odds, respectively, of the presence of a given medical condition. Relationships of race/ethnicity, education, service branch, and marital status with medical conditions were inconsistent. Finally, in exploratory factor analyses of health behaviors, associations of behavioral patterns—two patterns in particular—with medical conditions were associated with medical conditions as expected: the pattern characterized by a “healthy” diet was associated with lower odds of all medical conditions except high blood pressure, while the pattern characterized by “bad” habits (including lack of sleep) was associated with higher odds of every medical condition.

We observed robust associations between short sleep and each medical condition in the present study, ranging from 36% higher odds of overweight/obesity to over double the odds of hypertension, in contrast to inconsistent findings for diet and exercise, and smoking. There is increasing evidence of the importance of sleep to health [17–23], for example, a 2015 meta-analysis of 10 prospective cohort studies investigating sleep in relation to type 2 diabetes risk reported a U-shaped, dose-response relationship between sleep duration and diabetes risk [22]. The lowest risk was observed at sleep durations of 7–8 h/day, with incrementally higher risk of diabetes below or above that duration. Several reports have linked short sleep durations with higher risk of overweight/obesity [17, 20, 21], including an analysis of the representative U.S. population, in which authors reported that, relative to those who slept 7–8 h/day, very short sleepers (< 5 h) had 30% higher odds of being overweight and were twice as likely to be obese, while (5–6 h) short sleepers had 20% higher odds of being overweight and 57% higher odds of being obese [20]. In addition, a 2013 meta-analysis of 17 cohorts investigating the relationship between sleep duration and incident hypertension reported that short sleep duration conferred 21% higher risk of developing the condition [18]. Because recommendations to change diet, exercise, and smoking behaviors, but not sleep behaviors, are a part of many primary and secondary prevention recommendations [24–26], it is possible that individuals are more likely to make changes to these behaviors, rather than sleep, in an effort to prevent or as a result of receiving a diagnosis. In fact, sleep is not mentioned as a modifiable risk factor in disease prevention/management guidelines from the American Diabetes Association or from the American College of Cardiology/American Heart Association [24–26], and thus may not be a part of lifestyle changes individuals undertake to modify their perceived disease risk.

In military populations, the critical importance of sleep has only recently become part of the “Performance Triad”, an educational initiative introduced in 2013 on health readiness and wellness including 1) physical activity; 2) nutrition; and 3) sleep [27, 28]. Our data therefore confirm the importance of the inclusion of sleep in the “Triad” initiative. In our study, poor sleep may also be a complex product of poor health, and occupational or personal stress [29, 30]. Other studies have indicated that sleep disorders, including insomnia and sleep apnea, appear to be more common in service members than in civilians [31–33]. Our observations using self-reported survey data, on the strong relationship between poor sleep and behaviors and medical conditions, agree with other studies usingself-reported surveys of military personnel [34].

Our observations regarding select sociodemographic characteristics, notably age, sex, and marital status, as being associated with select health behaviors generally agree with existing evidence [2, 7]. With the exception of odds of high blood glucose, which was higher in females versus males, other associations of age and sex—well-known biological drivers of health risks—were as generally expected. Married individuals reported better health behaviors than never- or previously married individuals; however this did not necessarily translate into lower odds of having medical conditions for married individuals. Given the cross-sectional nature of the study design, we can only speculate as to why: first, married individuals may be more likely to seek routine medical care at the urging of their spouse [35–37], and thus have been told by a physician of a prevalent condition that would have otherwise gone undetected. Second, cross-sectional analyses preclude assessment of sequential behaviors; the reported behaviors may be in response to a diagnosis, a form of reverse causality. In addition, although analyses were adjusted for age and presence of children, as well as other sociodemographic characteristics, relationships between marital status and medical conditions are nuanced and complicated by time-related factors, including secular trends [37–39]. For example, a recent study using data from a panel of nationally representative households observed that while a protective association of long marriage on women’s self-reported health was evident in the earlier birth cohort (born 1955–1964), it had reversed in the most recent birth cohort (born 1975–1984) [39].

There were other unexpected associations. For example, we observed intake of snacks, sweets, and sugary drinks (in addition to the “Unhealthy diet” factor which included these foods) were associated with lower odds of overweight/obesity, which is generally the opposite of what would be expected given evidence regarding the long-term negative consequences of consuming these foods on health [40–43]. These associations held despite adjusting for history of weight loss and current enrollment in a weight control program. It is possible this finding reflects the generally young age of the respondents in this military population, or that some respondents were simply not at risk of overweight. This observation, in fact, appears to be consistent with cross-sectional nationally representative NHANES data on snacking and sugary beverages in both adolescents [44] and adults [45] which have shown no clearly discernible associations with overweight or obesity. In addition, state-based BRFSS 2013 data indicate that sugar-sweetened beverage intake declines with higher age. Intake was most prevalent among younger adults (age 18–24 y), at 43% reporting consuming these beverages one or more times per day, while those in older age groups reported less consumption (38% of those age 25–34 y, 30% in those age 35–54 y, and 19% in those age ≥ 55 y) [46]. On the other hand, our exploratory observations regarding the “healthy diet” pattern and favorable odds of medical conditions were consistent with dietary pattern literature [47–50].

As noted above, our analyses are limited by the cross-sectional nature of the survey, which precludes causal inferences. Particularly when assessing health behaviors and medical conditions concurrently, a behavioral pattern may reflect a subset of respondents who have changed their behaviors as result of having been alerted to a health risk [51–56]. Furthermore, we were limited to gross rather than granular assessments of dietary components. We were also likely limited in the validity of our inferences by social desirability response bias of self-reported data. In addition, non-response bias, length of the survey, and fear of loss of anonymity among either non-respondents or due to selective non-responses in those excluded from the present analysis may also limit our ability to make inferences or generalize our findings to the broader Armed Services, despite using revised sampling weights to accommodate exclusions.

## Conclusions

This study presents relationships between sociodemographic characteristics, health behaviors, and doctor-informed medical conditions and overweight/obesity in the U.S. Armed Forces, which are typically not included, or not separately reported, in national or state health surveys.

Despite aforementioned limitations and our inability to deduce causal relationships, we observed pronounced associations between health behaviors—especially sleep duration—and medical conditions, thus adding to evidence that sleep is a critical, potentially modifiable behavior within this population. It is recognized that there are times when sleep restriction is unavoidable in this population (e.g., during periodic training and mission scenarios), however, adequate sleep should continue to be promoted as an important part of overall health and wellness throughout the military community.

## Additional file

Additional file 1: **Table S1.** Unweighted, unadjusted characteristics of respondents who were included vs. excluded in the present analysis, from the 2011 Health Related Behaviors Survey. **Table S2.** Factors and factor loadings of 17 health behaviors derived from principal components analysis with varimax rotation. **Table S3.** Odds ratios (95% CI) of medical conditions by sociodemographic characteristics. **Table S4.** Description of Data: Odds ratios (95% CI) of medical conditions by health behaviors. (DOCX 50 kb)

## Abbreviations

BMI: Body mass index
CI: Confidence interval
d: Day
DOD: Department of Defense
HDL: High-density lipoprotein
HRBS: Health Related Behavior Survey
hrs: Hours
OR: Odds ratio
ser: Servings
TMA: Tricare Management Activity
U.S.: United States
USCG: United States Coast Guard
wk.: Week
y: Years
