# Supplementary material for: Self-reported health behaviors, including sleep, correlate with doctor-informed medical conditions: data from the 2011 Health Related Behaviors Survey of U.S. Active Duty Military Personnel
Source: BMC Public Health. 2018 Jul 11;18:853. doi: 10.1186/s12889-018-5781-2 (PMC6042384; doi:10.1186/s12889-018-5781-2)
Supplement: Supplementary file 1 — Table S1. Unweighted, unadjusted characteristics of respondents who were included vs. excluded in the present analysis, from the 2011 Health Related Behaviors Survey. Table S2. Factors and factor loadings of 17 health behaviors derived from principal components analysis with varimax rotation. Table S3. Odds ratios (95% CI) of medical conditions by sociodemographic characteristics. Table S4. Description of Data: Odds ratios (95% CI) of medical conditions by health behaviors. (DOCX 50 kb) [file 12889_2018_5781_MOESM1_ESM.docx]

**Additional files**

“Self-Reported Health Behaviors, Including Sleep, Correlate with Doctor-Informed Medical Conditions: Data from the 2011 Health Related Behaviors Survey of US Active Duty Military Personnel”

Adela Hruby, PhD, MPH, Harris Lieberman, PhD, Tracey J. Smith, PhD, RD

**Table S1.** Unweighted, unadjusted characteristics of respondents who were included vs. excluded in the present analysis, from the 2011 Health Related Behaviors Survey.

**Table S2.** Factors and factor loadings of 17 health behaviors derived from principal components analysis with varimax rotation.

**Table S3.** Odds ratios (95% CI) of medical conditions by sociodemographic characteristics.

**Table S4.** Odds ratios (95% CI) of medical conditions by health behaviors.

**Table S1.** Unweighted, unadjusted characteristics of respondents who were included versus excluded in the present analysis, from the 2011 Health Related Behaviors Survey.

|  | **Included (N=27,034)** | **Excluded (N=12,843)** | |
| --- | --- | --- | --- |
|  | **Mean±SD** | **N missing** | **Mean±SD*** |
| Children (N) | 0.9±1.2 | 93 | 0.8±1.2 |
| Age (yrs) | 31.97±8.36 | 4256 | 30.67±8.22 |
| Health risks (N of 5 possible) | 0.44±0.89 | 30 | 0.42±0.86 |
| BMI (kg/m^2^) | 25.9±3.3 | 148 | 25.8±3.3 |
| Moderate exercise (min/wk) | 167.8±125.4 | 117 | 170.5±128.4 |
| Vigorous exercise (min/wk) | 109.7±104.2 | 117 | 115.5±111.0 |
| Strength training (min/wk) | 94.1±105.3 | 117 | 105.4±114.1 |
| Sleep (hrs/24 hrs) | 6.4±1.3 | 8233 | 6.6±2.8 |
| Alcohol (drinks/day last month) | 0.27±0.50 | 1510 | 0.27±0.55 |
| *Dietary Intake* |  |  |  |
| Fruit (ser/wk) | 8.68±6.28 | 50 | 8.56±6.37 |
| Starch (ser/wk) | 5.71±4.82 | 50 | 5.92±5.11 |
| Vegetables (ser/wk) | 9.68±6.19 | 50 | 9.44±6.30 |
| Whole grains (ser/wk) | 9.22±6.20 | 50 | 9.07±6.30 |
| Dairy (ser/wk) | 9.31±6.16 | 50 | 8.96±6.26 |
| Lean meat (ser/wk) | 9.49±6.13 | 50 | 9.41±6.25 |
| Snacks (ser/wk) | 3.58±4.18 | 50 | 3.87±4.55 |
| Sweets (ser/wk) | 3.70±4.38 | 50 | 3.77±4.63 |
| Sugary drinks (ser/wk) | 4.55±5.74 | 50 | 4.97±6.01 |
| Caffeinated drinks (ser/wk) | 8.30±7.24 | 50 | 7.87±7.12 |
| Fried foods (ser/wk) | 2.57±3.38 | 50 | 2.97±3.90 |

*Of those who had these data, but were excluded because of other missing data.

**Table S2.** Factors and factor loadings of 17 health behaviors derived from principal components analysis with varimax rotation.*

|  | **Factor1** | **Factor2** | **Factor3** | **Factor4** |
| --- | --- | --- | --- | --- |
| **Health Behaviors** | "Healthy diet" | "Unhealthy diet" | "Exercise" | "Bad habits" |
| Moderate exercise (min/wk) | 0.11 | 0.01 | **0.75** | 0.03 |
| Vigorous exercise (min/wk) | 0.10 | -0.02 | **0.83** | -0.01 |
| Strength training (min/wk) | 0.13 | -0.06 | **0.80** | -0.01 |
| Sleep (hrs/24 hrs) | 0.06 | 0.03 | -0.04 | **-0.49** |
| Alcohol (drinks/d last month) | -0.04 | 0.02 | 0.04 | **0.60** |
| Smoking status | -0.04 | 0.03 | -0.01 | **0.74** |
| *Dietary variables (ser/wk)* |  |  |  |  |
| Fruit | **0.76** | -0.04 | 0.10 | -0.09 |
| Starch | **0.58** | **0.36** | 0.04 | 0.07 |
| Vegetables | **0.80** | -0.08 | 0.05 | 0.01 |
| Whole grains | **0.73** | 0.11 | 0.08 | -0.02 |
| Dairy | **0.63** | 0.14 | 0.03 | -0.05 |
| Lean meat | **0.73** | 0.003 | 0.17 | 0.01 |
| Snacks | 0.09 | **0.81** | -0.02 | 0.02 |
| Sweets | 0.11 | **0.75** | -0.06 | -0.11 |
| Sugary drinks | -0.03 | **0.69** | -0.001 | 0.18 |
| Caffeinated drinks | 0.18 | **0.24** | -0.14 | **0.50** |
| Fried food | 0.06 | **0.77** | 0.002 | 0.08 |
| *Variance explained* | 3.12 | 2.52 | 1.96 | 1.47 |
| *Total variance = 9.07* |  |  |  |  |

*Items in bold indicate >|0.20| factor loading, and contributed to factor naming.

**Table S3.** Odds ratios (95% CI) of medical conditions by sociodemographic characteristics (models 1 and 2).*

|  |  | **High blood pressure  (N cases = 3713)** | | **High cholesterol  (N cases = 4098)** | | **High triglycerides  (N cases = 1745)** | | |
| --- | --- | --- | --- | --- | --- | --- | --- | --- |
| **Demographic characteristic** | **Categories** | **Model 1** | **Model 2** | **Model 1** | **Model 2** | **Model 1** | **Model 2** |  |
| **Age (vs. ≤20 yrs)** | 21–25 yrs | 1.86 (1.24–2.80) | 1.69 (1.13–2.53) | 1.32 (0.71–2.48) | 1.27 (0.67–2.38) | 1.33 (0.44–4.08) | 1.25 (0.41–3.83) |  |
|  | 26–35 yrs | 2.67 (1.75–4.07) | 2.30 (1.51–3.50) | 3.17 (1.69–5.93) | 2.94 (1.57–5.50) | 2.85 (0.95–8.54) | 2.59 (0.87–7.74) |  |
|  | 36–45 yrs | 4.42 (2.86–6.84) | 3.77 (2.43–5.83) | 8.77 (4.63–16.61) | 8.12 (4.28–15.38) | 8.85 (2.93–26.74) | 7.94 (2.63–23.93) |  |
|  | 46+ yrs | 7.31 (4.63–11.55) | 6.33 (4.01–10.01) | 13.46 (7.02–25.82) | 12.54 (6.54–24.06) | 13.09 (4.29–40.00) | 11.81 (3.88–36.00) |  |
| **Service Branch (vs. Army)** | Air Force | 0.77 (0.68–0.88) | 0.80 (0.70–0.91) | 0.96 (0.85–1.10) | 0.98 (0.86–1.11) | 1.25 (1.04–1.51) | 1.26 (1.05–1.52) |  |
|  | Coast Guard | 0.68 (0.59–0.80) | 0.72 (0.62–0.85) | 1.20 (1.04–1.39) | 1.24 (1.07–1.44) | 1.54 (1.26–1.89) | 1.59 (1.29–1.95) |  |
|  | Marine Corps | 0.98 (0.84–1.14) | 0.98 (0.84–1.15) | 0.95 (0.81–1.12) | 0.95 (0.81–1.11) | 0.68 (0.52–0.88) | 0.68 (0.52–0.88) |  |
|  | Navy | 0.85 (0.73–0.99) | 0.85 (0.73–0.99) | 1.24 (1.07–1.43) | 1.24 (1.07–1.43) | 1.57 (1.29–1.91) | 1.57 (1.28–1.91) |  |
| **Sex (vs. male)** | Female | 0.54 (0.49–0.60) | 0.54 (0.49–0.60) | 0.65 (0.59–0.73) | 0.65 (0.58–0.72) | 0.57 (0.48–0.67) | 0.56 (0.47–0.66) |  |
| **Education** | Some college | 0.99 (0.86–1.13) | 0.99 (0.87–1.14) | 1.15 (0.98–1.33) | 1.15 (0.98–1.34) | 1.19 (0.95–1.49) | 1.18 (0.94–1.48) |  |
| **(vs. high school or equivalent)** | 2-year college | 0.83 (0.71–0.98) | 0.84 (0.71–0.99) | 1.13 (0.95–1.34) | 1.13 (0.95–1.34) | 1.33 (1.04–1.70) | 1.33 (1.03–1.70) |  |
|  | 4-year college | 0.82 (0.68–0.98) | 0.84 (0.69–1.00) | 1.18 (0.97–1.42) | 1.19 (0.98–1.43) | 1.59 (1.21–2.08) | 1.59 (1.22–2.08) |  |
|  | Beyond college | 0.87 (0.71–1.06) | 0.89 (0.72–1.08) | 1.37 (1.12–1.69) | 1.39 (1.13–1.71) | 1.72 (1.30–2.29) | 1.73 (1.30–2.30) |  |
| **Race/ethnicity** | Hispanic | 0.90 (0.77–1.04) | 0.89 (0.77–1.03) | 1.07 (0.93–1.24) | 1.06 (0.91–1.22) | 1.05 (0.85–1.29) | 1.04 (0.84–1.27) |  |
| **(vs. non-Hispanic white)** | Non-Hispanic black | 1.63 (1.42–1.87) | 1.68 (1.46–1.93) | 1.10 (0.94–1.28) | 1.11 (0.96–1.30) | 0.56 (0.43–0.73) | 0.58 (0.44–0.75) |  |
|  | Other/multiple | 1.40 (1.20–1.63) | 1.43 (1.23–1.67) | 1.42 (1.22–1.65) | 1.44 (1.23–1.67) | 1.34 (1.10–1.65) | 1.37 (1.12–1.67) |  |
| **Marital status** | Currently married | 1.12 (0.97–1.31) | 1.12 (0.97–1.31) | 1.26 (1.07–1.47) | 1.26 (1.08–1.48) | 1.02 (0.80–1.30) | 1.03 (0.81–1.30) |  |
| **(vs. never married)** | Separated/divorced/ widowed | 1.32 (1.10–1.58) | 1.31 (1.10–1.57) | 1.17 (0.97–1.41) | 1.18 (0.98–1.42) | 0.93 (0.70–1.23) | 0.93 (0.71–1.23) |  |
| **Children (vs. none)** | 1+ children | 1.06 (0.96–1.18) | 1.06 (0.96–1.18) | 1.08 (0.98–1.20) | 1.08 (0.98–1.20) | 1.24 (1.08–1.43) | 1.24 (1.08–1.43) |  |
| **Pay grade (vs. E1-E4)** | E5-E6 | 1.60 (1.38–1.87) | 1.57 (1.34–1.85) | 1.92 (1.61–2.31) | 1.90 (1.58–2.28) | 2.07 (1.58–2.71) | 2.10 (1.60–2.76) |  |
|  | E7-E9 | 1.65 (1.38–1.98) | 1.68 (1.39–2.03) | 2.05 (1.68–2.51) | 2.07 (1.68–2.55) | 2.10 (1.56–2.83) | 2.22 (1.64–3.00) |  |
|  | WO1-WO5 | 1.40 (1.09–1.79) | 1.41 (1.10–1.82) | 2.27 (1.76–2.93) | 2.28 (1.76–2.95) | 2.12 (1.47–3.06) | 2.23 (1.53–3.24) |  |
|  | O1-O3 | 1.09 (0.88–1.35) | 1.12 (0.90–1.39) | 1.40 (1.12–1.75) | 1.41 (1.13–1.77) | 1.35 (0.98–1.87) | 1.42 (1.03–1.97) |  |
|  | O4-O10 | 1.13 (0.89–1.44) | 1.15 (0.90–1.47) | 1.61 (1.26–2.06) | 1.63 (1.27–2.09) | 1.40 (0.99–1.98) | 1.49 (1.05–2.12) |  |

|  |  | **Low HDL cholesterol  (N cases = 1921)** | | **High blood glucose  (N cases = 499)** | | **Overweight/obesity  (N cases = 16292)** | | |
| --- | --- | --- | --- | --- | --- | --- | --- | --- |
| **Demographic characteristic** | **Categories** | **Model 1** | **Model 2** | **Model 1** | **Model 2** | **Model 1** | **Model 2** |  |
| **Age (vs. ≤20 yrs)** | 21–25 yrs | 1.27 (0.47–3.43) | 1.21 (0.45–3.25) | 1.23 (0.37–4.14) | 1.13 (0.34–3.79) | 1.61 (1.36–1.90) | 1.54 (1.28–1.84) |  |
|  | 26–35 yrs | 3.03 (1.14–8.06) | 2.79 (1.05–7.38) | 1.54 (0.45–5.25) | 1.34 (0.40–4.54) | 2.46 (2.05–2.97) | 2.21 (1.80–2.70) |  |
|  | 36–45 yrs | 7.60 (2.84–20.34) | 6.86 (2.57–18.32) | 4.07 (1.18–14.00) | 3.54 (1.04–12.06) | 4.19 (3.39–5.17) | 3.82 (3.05–4.78) |  |
|  | 46+ yrs | 12.92 (4.77–34.96) | 11.80 (4.37–31.88) | 11.52 (3.23–41.10) | 10.06 (2.85–35.52) | 4.63 (3.61–5.95) | 4.19 (3.22–5.45) |  |
| **Service Branch (vs. Army)** | Air Force | 0.96 (0.81–1.14) | 0.97 (0.82–1.15) | 0.72 (0.52–1.01) | 0.73 (0.52–1.02) | 0.77 (0.69–0.85) | 0.79 (0.71–0.88) |  |
|  | Coast Guard | 1.24 (1.02–1.49) | 1.27 (1.04–1.54) | 1.00 (0.68–1.45) | 1.01 (0.68–1.50) | 0.99 (0.88–1.11) | 1.05 (0.93–1.19) |  |
|  | Marine Corps | 0.66 (0.52–0.83) | 0.66 (0.53–0.83) | 1.02 (0.69–1.50) | 1.01 (0.69–1.50) | 0.80 (0.71–0.90) | 0.78 (0.69–0.88) |  |
|  | Navy | 1.26 (1.04–1.52) | 1.25 (1.04–1.51) | 1.45 (1.05–2.01) | 1.45 (1.05–2.02) | 1.01 (0.89–1.14) | 1.04 (0.91–1.18) |  |
| **Sex (vs. male)** | Female | 0.48 (0.41–0.56) | 0.47 (0.40–0.55) | 1.42 (1.09–1.84) | 1.35 (1.03–1.77) | 0.34 (0.31–0.36) | 0.29 (0.27–0.31) |  |
| **Education** | Some college | 1.24 (1.00–1.55) | 1.25 (1.00–1.56) | 0.95 (0.67–1.36) | 0.95 (0.66–1.36) | 1.05 (0.95–1.15) | 1.03 (0.93–1.14) |  |
| **(vs. high school or equivalent)** | 2-year college | 1.48 (1.16–1.89) | 1.50 (1.18–1.91) | 1.01 (0.68–1.52) | 1.01 (0.67–1.51) | 0.90 (0.79–1.02) | 0.88 (0.77–1.00) |  |
|  | 4-year college | 1.50 (1.16–1.94) | 1.52 (1.18–1.96) | 0.63 (0.40–0.99) | 0.62 (0.40–0.98) | 0.95 (0.83–1.10) | 0.94 (0.81–1.09) |  |
|  | Beyond college | 1.88 (1.41–2.50) | 1.92 (1.45–2.56) | 1.11 (0.68–1.82) | 1.11 (0.67–1.82) | 0.86 (0.73–1.01) | 0.83 (0.70–0.98) |  |
| **Race/ethnicity** | Hispanic | 0.91 (0.74–1.12) | 0.90 (0.73–1.10) | 1.41 (1.01–1.98) | 1.39 (1.00–1.95) | 1.47 (1.32–1.64) | 1.43 (1.28–1.59) |  |
| **(vs. non-Hispanic white)** | Non-Hispanic black | 0.76 (0.61–0.96) | 0.78 (0.62–0.98) | 1.35 (0.94–1.96) | 1.42 (0.99–2.05) | 1.26 (1.11–1.43) | 1.36 (1.20–1.55) |  |
|  | Other/multiple | 1.17 (0.95–1.43) | 1.20 (0.98–1.47) | 1.84 (1.32–2.57) | 1.90 (1.36–2.66) | 0.89 (0.79–1.00) | 0.91 (0.80–1.03) |  |
| **Marital status** | Currently married | 0.95 (0.76–1.20) | 0.96 (0.76–1.20) | 1.10 (0.71–1.69) | 1.11 (0.72–1.72) | 1.21 (1.10–1.33) | 1.25 (1.14–1.38) |  |
| **(vs. never married)** | Separated/divorced/ widowed | 0.96 (0.74–1.25) | 0.96 (0.74–1.25) | 1.41 (0.89–2.25) | 1.42 (0.89–2.26) | 1.04 (0.92–1.17) | 1.07 (0.94–1.21) |  |
| **Children (vs. none)** | 1+ children | 1.32 (1.16–1.51) | 1.32 (1.16–1.52) | 1.63 (1.26–2.11) | 1.64 (1.27–2.11) | 1.23 (1.14–1.34) | 1.23 (1.13–1.34) |  |
| **Pay grade (vs. E1-E4)** | E5-E6 | 1.87 (1.43–2.45) | 1.86 (1.41–2.46) | 1.48 (1.00–2.19) | 1.63 (1.09–2.45) | 1.33 (1.21–1.47) | 1.54 (1.39–1.72) |  |
|  | E7-E9 | 2.08 (1.55–2.78) | 2.13 (1.57–2.89) | 1.21 (0.79–1.85) | 1.41 (0.90–2.21) | 1.38 (1.20–1.58) | 1.73 (1.50–2.01) |  |
|  | WO1-WO5 | 2.21 (1.54–3.17) | 2.25 (1.55–3.26) | 0.87 (0.49–1.54) | 1.00 (0.55–1.81) | 1.50 (1.20–1.87) | 1.87 (1.49–2.35) |  |
|  | O1-O3 | 1.30 (0.94–1.79) | 1.34 (0.96–1.86) | 0.90 (0.53–1.52) | 1.05 (0.61–1.80) | 0.93 (0.80–1.08) | 1.18 (1.01–1.37) |  |
|  | O4-O10 | 1.54 (1.09–2.19) | 1.58 (1.11–2.27) | 0.72 (0.41–1.26) | 0.86 (0.48–1.54) | 0.98 (0.81–1.19) | 1.27 (1.04–1.55) |  |

*Logistic regressions were weighted by sample weights. Model 1 includes all sociodemographic characteristics simultaneously. Model 2 is additionally adjusted for current enrollment in a weight-loss program, history of weight loss, and history of deployment.

**Table S4.** Odds ratios (95% CI) of medical conditions by health behaviors.*

|  | **High blood pressure (N cases = 3713)** | | | **High cholesterol (N cases = 4098)** | | |
| --- | --- | --- | --- | --- | --- | --- |
| **Heath Behavior** | **Model 1** | **Model 2** | **Model 3** | **Model 1** | **Model 2** | **Model 3** |
| **Dietary Intake (per ser/wk)** | |  |  |  |  |  |
| Fruit | 1.00 (0.99–1.01) | 1.00 (0.99–1.01) | 1.00 (0.99–1.01) | 1.00 (0.99–1.01) | 1.00 (0.99–1.01) | 1.00 (0.99–1.01) |
| Starch | 0.99 (0.98–1.00) | 0.99 (0.98–1.00) | 0.99 (0.98–1.00) | 0.99 (0.98–1.00) | 0.99 (0.98–1.00) | 0.99 (0.98–1.00) |
| Vegetables | 1.01 (1.00–1.02) | 1.00 (0.99–1.01) | 1.00 (0.99–1.01) | 1.01 (1.01–1.02) | 1.00 (0.99–1.01) | 1.00 (0.99–1.01) |
| Whole grains | 0.99 (0.98–1.00) | 1.00 (0.99–1.01) | 1.00 (0.99–1.01) | 0.99 (0.98–1.00) | 1.00 (0.99–1.01) | 1.00 (0.99–1.01) |
| Dairy | 0.99 (0.99–1.00) | 1.00 (0.99–1.01) | 1.00 (0.99–1.01) | 0.98 (0.97–0.99) | 0.99 (0.98–1.00) | 0.99 (0.98–1.00) |
| Lean meat | 0.98 (0.98–0.99) | 0.99 (0.98–1.00) | 0.99 (0.98–1.00) | 0.98 (0.97–0.99) | 0.99 (0.98–1.00) | 0.99 (0.98–1.00) |
| Snacks | 0.99 (0.98–1.01) | 1.00 (0.98–1.01) | 1.00 (0.98–1.02) | 0.98 (0.97–0.99) | 0.99 (0.98–1.00) | 0.99 (0.98–1.00) |
| Sweets | 0.99 (0.97–1.00) | 0.99 (0.97–1.00) | 0.99 (0.97–1.00) | 1.01 (1.00–1.02) | 1.00 (0.99–1.02) | 1.00 (0.99–1.02) |
| Sugary drinks | 0.99 (0.98–1.00) | 1.00 (0.99–1.01) | 1.00 (0.99–1.01) | 0.98 (0.97–0.99) | 1.00 (0.99–1.01) | 1.00 (0.99–1.01) |
| Caffeinated drinks | 1.02 (1.01–1.03) | 1.00 (1.00–1.01) | 1.00 (1.00–1.01) | 1.04 (1.03–1.04) | 1.00 (0.99–1.01) | 1.00 (0.99–1.01) |
| Fried food | 1.01 (0.99–1.02) | 1.02 (1.00–1.04) | 1.02 (1.00–1.04) | 0.99 (0.98–1.01) | 1.02 (1.00–1.03) | 1.02 (1.00–1.04) |
| **Exercise (per 15 min/wk)** |  |  |  |  |  |  |
| Moderate exercise | 1.00 (0.99–1.00) | 1.00 (0.99–1.01) | 1.00 (0.99–1.01) | 1.00 (0.99–1.00) | 1.00 (1.00–1.01) | 1.00 (1.00–1.01) |
| Vigorous exercise | 1.00 (0.99–1.00) | 1.00 (0.99–1.00) | 0.99 (0.99–1.00) | 0.99 (0.99–1.00) | 0.99 (0.99–1.00) | 0.99 (0.99–1.00) |
| Strength training | 1.00 (0.99–1.00) | 1.00 (0.99–1.01) | 1.00 (0.99–1.01) | 0.97 (0.96–0.98) | 0.98 (0.97–0.99) | 0.98 (0.97–0.99) |
| **Smoking (vs. Never)** |  |  |  |  |  |  |
| Former | 1.36 (1.22–1.51) | 1.26 (1.13–1.42) | 1.26 (1.12–1.41) | 1.19 (1.08–1.32) | 1.11 (0.99–1.24) | 1.10 (0.99–1.24) |
| Current | 1.15 (1.02–1.29) | 1.33 (1.17–1.51) | 1.31 (1.16–1.49) | 0.74 (0.65–0.83) | 1.02 (0.90–1.17) | 1.01 (0.89–1.16) |
| **Alcohol (per drink/day)** | 1.12 (1.04–1.21) | 1.19 (1.10–1.29) | 1.20 (1.11–1.29) | 0.93 (0.85–1.01) | 1.04 (0.95–1.14) | 1.04 (0.95–1.14) |
| **Sleep (vs. 7 to <8 hrs/night)** | |  |  |  |  |  |
| <5 hrs/night | 2.35 (2.02–2.74) | 2.30 (1.96–2.70) | 2.22 (1.89–2.61) | 1.28 (1.09–1.50) | 1.40 (1.18–1.66) | 1.37 (1.16–1.63) |
| 5 to <6 hrs/night | 1.51 (1.32–1.73) | 1.46 (1.27–1.68) | 1.43 (1.25–1.65) | 1.19 (1.05–1.35) | 1.22 (1.06–1.39) | 1.20 (1.05–1.38) |
| 6 to <7 hrs/night | 1.26 (1.12–1.42) | 1.21 (1.07–1.36) | 1.19 (1.05–1.34) | 1.05 (0.94–1.17) | 1.02 (0.91–1.14) | 1.01 (0.90–1.13) |
| 8 to <9 hrs/night | 0.82 (0.69–0.97) | 0.92 (0.78–1.09) | 0.92 (0.78–1.10) | 0.81 (0.69–0.94) | 0.99 (0.84–1.16) | 0.98 (0.83–1.16) |
| 9 to <10 hrs/night | 0.65 (0.43–0.98) | 0.86 (0.56–1.31) | 0.85 (0.56–1.30) | 0.63 (0.38–1.02) | 0.99 (0.59–1.66) | 0.99 (0.58–1.67) |
| 10+ hrs/night | 0.83 (0.49–1.40) | 1.16 (0.68–1.98) | 1.13 (0.66–1.94) | 0.74 (0.42–1.30) | 1.48 (0.81–2.70) | 1.43 (0.78–2.62) |

|  | **High triglycerides (N cases = 1745)** | | | **High blood glucose (N cases = 499)** | | |
| --- | --- | --- | --- | --- | --- | --- |
| **Heath Behavior** | **Model 1** | **Model 2** | **Model 3** | **Model 1** | **Model 2** | **Model 3** |
| **Dietary Intake (per ser/wk)** | |  |  |  |  |  |
| Fruit | 1.00 (0.99–1.02) | 1.00 (0.99–1.01) | 1.00 (0.99–1.01) | 0.99 (0.97–1.02) | 0.99 (0.96–1.01) | 0.99 (0.96–1.01) |
| Starch | 0.98 (0.97–1.00) | 0.98 (0.96–1.00) | 0.98 (0.96–1.00) | 0.99 (0.96–1.02) | 1.00 (0.97–1.03) | 1.00 (0.97–1.03) |
| Vegetables | 1.02 (1.01–1.03) | 1.00 (0.99–1.02) | 1.00 (0.99–1.02) | 1.00 (0.97–1.02) | 0.98 (0.96–1.01) | 0.99 (0.96–1.01) |
| Whole grains | 0.99 (0.98–1.01) | 1.00 (0.98–1.01) | 1.00 (0.98–1.01) | 1.00 (0.98–1.03) | 1.01 (0.98–1.03) | 1.01 (0.99–1.03) |
| Dairy | 0.99 (0.98–1.00) | 1.00 (0.99–1.01) | 1.00 (0.99–1.01) | 0.97 (0.95–1.00) | 0.98 (0.96–1.01) | 0.98 (0.96–1.01) |
| Lean meat | 0.98 (0.97–0.99) | 0.99 (0.97–1.00) | 0.99 (0.97–1.00) | 1.02 (0.99–1.04) | 1.03 (1.00–1.05) | 1.02 (1.00–1.05) |
| Snacks | 0.98 (0.96–1.00) | 0.99 (0.97–1.01) | 0.99 (0.97–1.01) | 0.96 (0.91–1.00) | 0.97 (0.93–1.02) | 0.97 (0.93–1.02) |
| Sweets | 1.01 (1.00–1.03) | 1.01 (0.99–1.03) | 1.01 (0.99–1.03) | 1.00 (0.96–1.03) | 0.99 (0.96–1.02) | 0.99 (0.96–1.03) |
| Sugary drinks | 0.97 (0.95–0.98) | 0.99 (0.98–1.01) | 0.99 (0.98–1.01) | 0.96 (0.93–0.99) | 0.97 (0.95–1.00) | 0.98 (0.95–1.00) |
| Caffeinated drinks | 1.04 (1.03–1.05) | 1.00 (0.99–1.01) | 1.00 (0.99–1.01) | 1.03 (1.01–1.05) | 1.01 (0.99–1.03) | 1.01 (0.99–1.03) |
| Fried food | 0.99 (0.97–1.01) | 1.01 (0.99–1.04) | 1.01 (0.99–1.04) | 1.02 (0.98–1.07) | 1.04 (1.00–1.08) | 1.04 (1.00–1.08) |
| **Exercise (per 15 min/wk)** |  |  |  |  |  |  |
| Moderate exercise | 0.99 (0.99–1.00) | 1.00 (0.99–1.01) | 1.00 (0.99–1.01) | 0.99 (0.97–1.00) | 0.99 (0.98–1.01) | 0.99 (0.98–1.01) |
| Vigorous exercise | 0.99 (0.98–1.00) | 0.99 (0.98–1.00) | 0.99 (0.98–1.00) | 0.98 (0.96–1.00) | 0.99 (0.97–1.01) | 0.98 (0.96–1.00) |
| Strength training | 0.96 (0.95–0.97) | 0.98 (0.96–0.99) | 0.98 (0.96–0.99) | 0.97 (0.95–0.99) | 0.98 (0.96–1.00) | 0.98 (0.96–1.01) |
| **Smoking (vs. Never)** |  |  |  |  |  |  |
| Former | 1.37 (1.18–1.59) | 1.31 (1.12–1.53) | 1.30 (1.11–1.52) | 1.17 (0.89–1.54) | 1.10 (0.82–1.45) | 1.10 (0.83–1.47) |
| Current | 0.91 (0.77–1.08) | 1.36 (1.14–1.63) | 1.35 (1.12–1.61) | 0.88 (0.65–1.19) | 1.04 (0.75–1.45) | 1.03 (0.75–1.43) |
| **Alcohol (per drink/day)** | 0.87 (0.76–0.99) | 0.99 (0.87–1.14) | 1.00 (0.87–1.14) | 0.88 (0.68–1.15) | 1.02 (0.80–1.30) | 1.03 (0.81–1.30) |
| **Sleep (vs. 7 to <8 hrs/night)** | |  |  |  |  |  |
| <5 hrs/night | 1.28 (1.03–1.59) | 1.55 (1.23–1.95) | 1.51 (1.20–1.91) | 2.57 (1.78–3.72) | 2.24 (1.52–3.30) | 2.18 (1.48–3.21) |
| 5 to <6 hrs/night | 1.15 (0.96–1.37) | 1.23 (1.02–1.48) | 1.22 (1.01–1.47) | 1.87 (1.34–2.61) | 1.67 (1.19–2.36) | 1.63 (1.16–2.29) |
| 6 to <7 hrs/night | 1.14 (0.97–1.33) | 1.15 (0.98–1.35) | 1.14 (0.97–1.33) | 1.21 (0.89–1.66) | 1.12 (0.82–1.53) | 1.09 (0.79–1.49) |
| 8 to <9 hrs/night | 0.78 (0.62–0.98) | 0.96 (0.76–1.21) | 0.95 (0.75–1.20) | 0.84 (0.55–1.28) | 0.94 (0.61–1.45) | 0.93 (0.60–1.44) |
| 9 to <10 hrs/night | 0.38 (0.17–0.88) | 0.61 (0.26–1.42) | 0.60 (0.26–1.41) | 0.54 (0.13–2.29) | 0.65 (0.15–2.77) | 0.65 (0.15–2.78) |
| 10+ hrs/night | 0.89 (0.42–1.89) | 1.97 (0.90–4.29) | 1.92 (0.87–4.22) | 0.66 (0.20–2.17) | 0.75 (0.23–2.46) | 0.66 (0.20–2.18) |

|  | **Low HDL cholesterol (N cases = 1921)** | | | **Overweight/obesity (N cases = 16292)** | | |
| --- | --- | --- | --- | --- | --- | --- |
| **Heath Behavior** | **Model 1** | **Model 2** | **Model 3** | **Model 1** | **Model 2** | **Model 3** |
| **Dietary Intake (per ser/wk)** | |  |  |  |  |  |
| Fruit | 1.01 (0.99–1.02) | 1.00 (0.99–1.02) | 1.00 (0.99–1.02) | 1.00 (0.99–1.00) | 1.00 (0.99–1.01) | 1.00 (0.99–1.01) |
| Starch | 1.00 (0.99–1.02) | 1.00 (0.99–1.02) | 1.00 (0.99–1.02) | 1.00 (0.99–1.01) | 0.99 (0.98–1.00) | 1.00 (0.99–1.00) |
| Vegetables | 1.01 (1.00–1.03) | 1.00 (0.98–1.01) | 1.00 (0.98–1.01) | 1.01 (1.00–1.01) | 1.00 (0.99–1.01) | 1.00 (0.99–1.01) |
| Whole grains | 1.00 (0.99–1.01) | 1.01 (1.00–1.02) | 1.01 (1.00–1.02) | 0.99 (0.98–0.99) | 0.99 (0.98–1.00) | 0.99 (0.98–1.00) |
| Dairy | 0.98 (0.97–0.99) | 0.99 (0.98–1.00) | 0.99 (0.98–1.00) | 0.99 (0.99–1.00) | 1.00 (0.99–1.00) | 1.00 (0.99–1.01) |
| Lean meat | 0.98 (0.96–0.99) | 0.99 (0.97–1.00) | 0.99 (0.97–1.00) | 1.00 (1.00–1.01) | 1.01 (1.00–1.02) | 1.00 (1.00–1.01) |
| Snacks | 0.98 (0.97–1.00) | 0.99 (0.97–1.02) | 0.99 (0.97–1.02) | 0.98 (0.97–0.99) | 0.99 (0.98–1.00) | 0.99 (0.98–1.00) |
| Sweets | 1.01 (1.00–1.03) | 1.01 (0.99–1.02) | 1.01 (0.99–1.03) | 0.98 (0.98–0.99) | 0.99 (0.98–1.00) | 0.99 (0.98–1.00) |
| Sugary drinks | 0.98 (0.96–0.99) | 1.00 (0.99–1.01) | 1.00 (0.99–1.01) | 0.98 (0.98–0.99) | 0.98 (0.98–0.99) | 0.98 (0.98–0.99) |
| Caffeinated drinks | 1.04 (1.03–1.05) | 1.00 (0.99–1.01) | 1.00 (0.99–1.01) | 1.03 (1.02–1.03) | 1.01 (1.00–1.01) | 1.01 (1.00–1.01) |
| Fried food | 0.97 (0.95–0.99) | 0.99 (0.97–1.02) | 0.99 (0.97–1.02) | 0.99 (0.97–1.00) | 0.99 (0.98–1.00) | 1.00 (0.98–1.01) |
| **Exercise (per 15 min/wk)** |  |  |  |  |  |  |
| Moderate exercise | 0.99 (0.98–1.00) | 1.00 (1.00–1.00) | 1.00 (0.99–1.01) | 1.00 (1.00–1.00) | 1.00 (0.99–1.00) | 1.00 (0.99–1.00) |
| Vigorous exercise | 0.99 (0.97–1.00) | 1.00 (1.00–1.00) | 0.98 (0.97–0.99) | 1.00 (1.00–1.00) | 1.00 (1.00–1.01) | 1.00 (0.99–1.01) |
| Strength training | 0.97 (0.96–0.98) | 1.00 (1.00–1.00) | 0.99 (0.98–1.00) | 1.00 (0.99–1.00) | 1.01 (1.01–1.02) | 1.02 (1.01–1.02) |
| **Smoking (vs. Never)** |  |  |  |  |  |  |
| Former | 1.23 (1.06–1.41) | 1.20 (1.03–1.40) | 1.20 (1.03–1.40) | 1.00 (1.00–1.01) | 1.15 (1.05–1.26) | 1.17 (1.06–1.28) |
| Current | 0.86 (0.73–1.00) | 1.29 (1.08–1.54) | 1.28 (1.07–1.52) | 1.01 (1.01–1.02) | 1.01 (0.92–1.11) | 0.99 (0.90–1.10) |
| **Alcohol (per drink/day)** | 0.89 (0.77–1.03) | 0.99 (0.84–1.15) | 0.99 (0.84–1.15) | 1.03 (0.97–1.10) | 1.04 (0.97–1.12) | 1.06 (0.99–1.14) |
| **Sleep (vs. 7 to <8 hrs/night)** | |  |  |  |  |  |
| <5 hrs/night | 1.31 (1.06–1.62) | 1.59 (1.26–1.99) | 1.55 (1.24–1.95) | 1.43 (1.26–1.62) | 1.42 (1.24–1.63) | 1.36 (1.18–1.57) |
| 5 to <6 hrs/night | 1.20 (1.01–1.42) | 1.29 (1.08–1.55) | 1.28 (1.07–1.53) | 1.30 (1.18–1.43) | 1.28 (1.15–1.42) | 1.26 (1.13–1.40) |
| 6 to <7 hrs/night | 1.11 (0.96–1.29) | 1.11 (0.95–1.30) | 1.10 (0.95–1.29) | 1.28 (1.18–1.39) | 1.23 (1.12–1.34) | 1.20 (1.10–1.32) |
| 8 to <9 hrs/night | 0.81 (0.65–1.01) | 1.02 (0.81–1.27) | 1.02 (0.81–1.27) | 0.87 (0.79–0.97) | 0.97 (0.87–1.09) | 0.98 (0.87–1.10) |
| 9 to <10 hrs/night | 0.37 (0.14–1.00) | 0.59 (0.22–1.60) | 0.58 (0.21–1.59) | 0.64 (0.50–0.83) | 0.87 (0.66–1.16) | 0.85 (0.64–1.13) |
| 10+ hrs/night | 0.47 (0.18–1.24) | 1.01 (0.39–2.65) | 0.98 (0.37–2.59) | 0.95 (0.68–1.33) | 1.35 (0.95–1.94) | 1.29 (0.88–1.89) |

*Logistic regressions were weighted by sample weights. Model 1 was adjusted for all health behaviors simultaneously. Model 2 was additionally adjusted for all sociodemographic characteristics (see Table 1). Model 3 was additionally adjusted for current enrollment in a weight-loss program, history of weight loss, and history of deployment.

*Note that for visualization in Figure 1A–F, dietary intake was converted to units of ser/day by multiplying the beta coefficient and standard error for the ser/wk estimate by 7, while exercise was converted to units of 30 min/wk by multiplying the beta coefficient and standard error for the min/wk estimate by 30 (or 15, in the above table). These conversions assume a linear relationship, and while beta estimates increase by the multiplicative factor, the significance of the estimate does not change (i.e., if confidence intervals cross 1 [the null value] in the original estimate, confidence intervals cross 1 in the multiplied estimate as well).*
